# The Critical Role of Fillers in Composite Polymer Electrolytes for Lithium Battery

**DOI:** 10.1007/s40820-023-01051-3

**Published:** 2023-03-28

**Authors:** Xueying Yang, Jiaxiang Liu, Nanbiao Pei, Zhiqiang Chen, Ruiyang Li, Lijun Fu, Peng Zhang, Jinbao Zhao

**Affiliations:** 1 College of Energy, Xiamen University, Xiamen, 361102 People’s Republic of China; 2 State Key Laboratory of Physical Chemistry of Solid Surfaces, Collaborative Innovation Centre of Chemistry for Energy Materials, State-Province Joint Engineering Laboratory of Power Source Technology for New Energy Vehicle, Engineering Research Center of Electrochemical Technology, Ministry of Education, College of Chemistry and Chemical Engineering, Xiamen University, Xiamen, 361005 People’s Republic of China; 3 College of Energy, Nanjing Technical University, Nanjing, 211816 People’s Republic of China

**Keywords:** Composite polymer electrolytes, Fillers, Ionic conductivity, Electrode–electrolyte interface

## Abstract

With excellent energy densities and highly safe performance, solid-state lithium batteries (SSLBs) have been hailed as promising energy storage devices. Solid-state electrolyte is the core component of SSLBs and plays an essential role in the safety and electrochemical performance of the cells. Composite polymer electrolytes (CPEs) are considered as one of the most promising candidates among all solid-state electrolytes due to their excellent comprehensive performance. In this review, we briefly introduce the components of CPEs, such as the polymer matrix and the species of fillers, as well as the integration of fillers in the polymers. In particular, we focus on the two major obstacles that affect the development of CPEs: the low ionic conductivity of the electrolyte and high interfacial impedance. We provide insight into the factors influencing ionic conductivity, in terms of macroscopic and microscopic aspects, including the aggregated structure of the polymer, ion migration rate and carrier concentration. In addition, we also discuss the electrode–electrolyte interface and summarize methods for improving this interface. It is expected that this review will provide feasible solutions for modifying CPEs through further understanding of the ion conduction mechanism in CPEs and for improving the compatibility of the electrode–electrolyte interface.

## Highlights

- The mechanism of the change in lithium-ion transport behavior caused by the incorporation of inorganic fillers into the polymer matrix is reviewed.
- The intrinsic factors of inorganic fillers to enhance the ionic conductivity of composite polymer electrolyte (CPEs) are investigated in depth.
- The contribution of inorganic fillers to inhibit dendrite growth and side reactions in CPEs is summarized.

## Graphical abstract

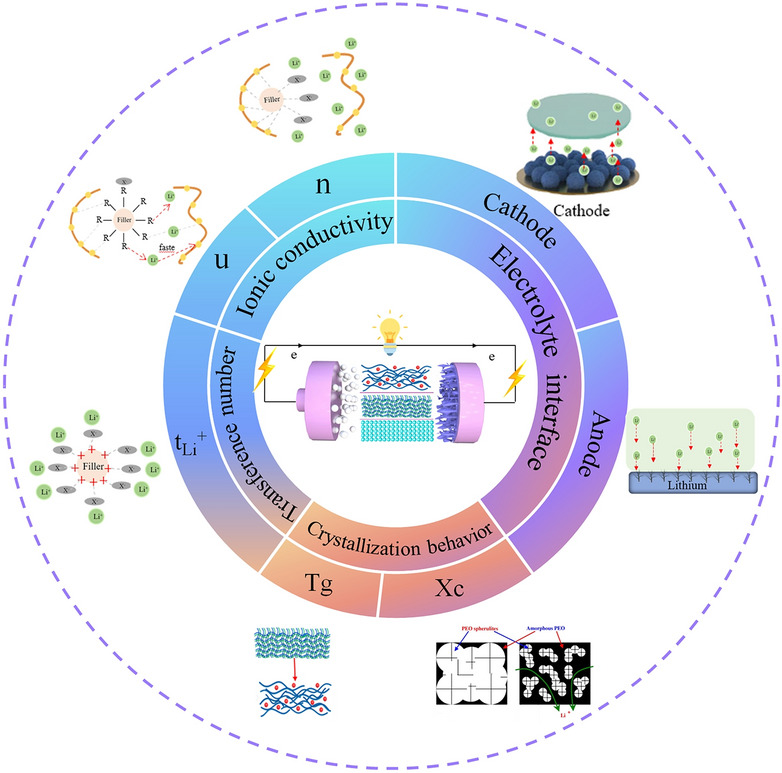

## Introduction

Traditional liquid electrolytes are used with safety issues such as flammability and leakage. Replacing liquid electrolytes with solid-state electrolytes is expected to fundamentally solve the safety problems of lithium-ion batteries [1, 2]. Moreover, solid-state electrolytes exhibit excellent mechanical strength and chemical neutrality, which can reduce the side reactions with lithium metal and inhibit the growth of lithium dendrites [3, 4]. Therefore, solid-state electrolytes are considered as a promising route for the preparation of lithium batteries with high safety performance, high stability and high energy density [5, 6].

To date, the solid-state electrolytes have been divided into three categories: solid polymer electrolytes (SPEs), inorganic solid electrolytes (ISEs), and composite polymer electrolytes (CPEs) [7]. Solid-state electrolytes should exhibit high ionic conductivity, a broad electrochemical window, an outstanding lithium-ion transference number (t_Li_^+^), enough mechanical strength, and great electrode compatibility [8]. ISEs, such as oxide electrolytes (garnet, NASICON, perovskite), sulfide electrolytes (Li_10_GeP_2_S_12_, Li_2_S–P_2_S_5_, Li_6_PS_5_X) and halide electrolytes (Li_3_YCl_6_, Li_3_ScCl_6_, Li_3_YBr_6_), have been widely investigated [9, 10]. ISEs show high mechanical robustness and excellent conductivity, which is even equal to that of liquid electrolytes. However, the commercial application of ISEs is limited by drawbacks such as poor electrode–electrolyte interfaces and processing properties. In contrast, SPEs with good flexibility can solve interface compatibility and processing problems [11, 12]. Due to the good solid–solid contact, the electrolyte can be well fitted to lithium metal for high-performance batteries. Many typical SPEs have been extensively studied, such as polyacrylonitrile (PAN) [13], poly (vinylidene fluoride‐hexafluoropropylene) (PVDF‐HFP) [14], polyethylene oxide (PEO) [15], and poly(ethylene glycol) dimethacrylate (PEGDMA) [16]. However, SPEs always suffer from poor ionic conductivity and low voltage tolerance.

CPEs, which consist of polymers, inorganic fillers and lithium salts, not only succeed in the virtues of processability and flexibility of SPE, but also bridge the discrepancy between SPE and ISEs by incorporating fillers [17]. Usually, the amount of filler is different in CPEs. When the filler content is lower than 50%, the filler can be approximately considered as being incorporated into the polymer. Otherwise, the polymer can be regarded as being incorporated into the filler. In recent years, CPEs have attracted much attention for their excellent electrochemical and safety properties [18–21]. However, in practical applications, CPEs cannot support the high-performance SSLBs, due to disappointing ionic conductivity and interfacial stability. Consequently, it is necessary to adopt some strategies to enhance the ionic conductivity and alleviate the interfacial issues of CPEs [19, 22].

Surprisingly, the inorganic fillers have an important effect on several properties of CPEs. Inorganic fillers can be divided into two categories: passive fillers and active fillers. Generally, active fillers (perovskite, garnet, LISICON, etc.), which can form continuous ion channels in the bulk phase and facilitate fast-ion transport, have a superior ionic conductivity. Li_3x_La_(2/3−x)_TiO_3_ (LLTO) is a representative active filler with a high ionic conductivity of 10^–3^ S cm^−1^ [15, 23, 24]. In regard to passive fillers, SiO_2_, Al_2_O_3_, TiO_2_, MgO and ZnO are the most researched. These fillers do not possess ion transport capabilities [25]. Nevertheless, the enhancement of the ionic conductivity of CPEs with passive fillers depends on the filler–polymer interface.

Thanks to the extensive studies of CPEs doped with different fillers, a fundamental understanding of the ion transport mechanisms in CPEs has been obtained. Inorganic fillers can disrupt the aggregated structure of the polymer matrix, reduce the crystallinity and increase the number of polymer chain segments that can be conducted [26]. Meanwhile, structural design and surface modification of inorganic fillers can facilitate the dissociation of lithium salts or establish new ion conduction channels. For example, some vertically aligned structures can minimize the distance of ion movement. The functional groups on the surface of the inorganic fillers will also have an effect on the carrier concentration in CPEs and the motion of polymer chains. Therefore, many factors of the filler can affect the performance of CPEs [27]. These changes in performance are reflected in the intrinsic ion transport. This interaction is mainly attributed to two categories: filler–polymer and filler–lithium salt. In CPEs, ion transport is dominated by polymer chains. Therefore, filler size, concentration and hybridization strategies are key steps in the fabrication of high-performance CPEs. In addition, some fillers can optimize the electrode–electrolyte interface through synergistic effects and reduce the ion transport resistance at the interface [28]. For example, good chemical stability can be matched with high-voltage cathode materials, and excellent mechanical strength can effectively inhibit the growth of lithium dendrites [29]. In addition, the internal Lewis acid–base interaction induces the uniform deposition of lithium ions and uniform ion transport flux and reduces the large accumulation of charges at the electrode–electrolyte.

In this review, we first introduce the composition of CPEs, including polymer matrix and species of fillers. Second, the contribution of fillers in CPEs is presented in terms of the bulk phase and interface. Regarding the bulk phase, the interactions are focused on the filler–polymer and filler–lithium salt. The former mainly affects the aggregated state structure of the polymer, as reflected by the changes in the crystallinity (Xc), glass transition temperature (Tg) and spherulite morphology of CPEs. The latter influences the ionic conductivity, and t_Li_^+^. From the perspective of the basic theory of physical chemistry, all of these factors are responsible for the ionic conductivity. For the electrode–electrolyte interface, the contributions of inorganic fillers at the cathode–electrolyte and anode–electrolyte interface are summarized. Both lowering the HOMO energy level of the CPEs and inducing uniform lithium deposition can effectively regulate the interfacial compatibility. Finally, we offer some suggestions for the development of CPEs with the hope of promoting the industrialization of high-performance solid-state lithium batteries.

## Overview of Composite Polymer Electrolytes

### Polymer Matrices

**Fig. 1:**
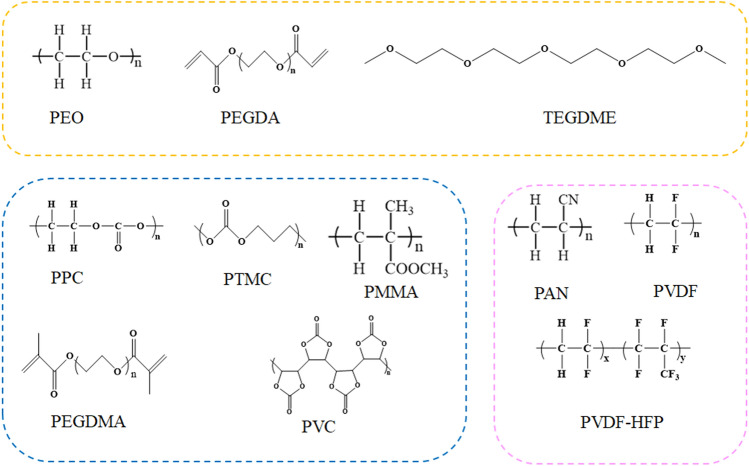
Chemical structure of commonly polymers [45–64]

Polymer electrolytes have been studied for many years. In 1973, Wright et al. [30] revealed that PEO with alkali metal salts possesses ionic conductivity. This finding set a precedent for the development of ion-conducting polymer. PEO, as a typical ion-conducting polymer, contains abundant ether-oxygen groups that can dissolve lithium salts and form complexes with lithium ions [31, 32]. In SPEs, lithium salts and polymers form complexes. Under this condition, the driving force of propulsion generated through the movement of the amorphous polymer chains promotes the jumping of anions and cations at the adjacent coordination sites. Directional motion, which is referred to as an ion-conducting process, is achieved under the external electric field. Therefore, it is generally agreed that ionic conduction mainly happens in the amorphous region of the polymer. Most ion-conducting polymers are semicrystalline at RT, including PAN, polyvinyl carbonate (PVC), polyvinylidene fluoride (PVDF), PVDF-HFP, polymethyl methacrylate (PMMA), polyethylene (glycol) diacrylate (PEGDA), tetraethylene glycol dimethacrylate (TEGDMA), and tetraethylene glycol dimethyl ether (TEGDME) [33, 34]. Common polymer matrices and their chemical structures are summarized in Fig. 1. And the molecular weight of common polymers is listed in Table 1. Due to the semicrystalline nature of these polymers, chain segment movement is difficult at RT. The ionic conductivity of these polymers at RT ranges from only 10^–6^ to 10^–8^ S cm^−1^ [35]. The addition of hydrogen bonds or *π*-conjugated groups in polymer chains is an effective way to enhance the ionic conductivity [36, 37]. Hydrogen bonding can occur through interactions with polar groups to relieve the coordination of strong polar groups with lithium ions to increase the carrier concentration. The *π*-conjugated groups can form new ion conduction channels [38].

However, a few studies have suggested that crystalline polymers can also conduct lithium ions [39, 40]. In contrast to conventional ion conduction, lithium-ion movement in crystalline polymers does not depend on relaxed segments, but on jumps in helical channels. PEO chains fold in an ordered framework to form an interlocking cylinder (channels). Lithium ions are present in the channels and the anions are located outside [41]. In addition to PEO, some plastic crystals are also attracting attention. The plastic crystals are a kind of material with a disordered direction and ordered position due to the rotational motion of molecules or ions at a certain temperature, such as succinonitrile (SN) and sebaconitrile. Because of the special structure, plastic crystals have excellent plasticity and diffusion rate. As a result, this type of solid-state electrolyte has a high ionic conductivity. SN, as a typical molecular plastic crystal, exhibits plastic crystal behavior at − 35∼62 °C [42]. Below − 35 °C, the SN molecule exists only in gauche conformation and all rotational motions are frozen. In contrast, the orientation disorder of the plastic phase of SN at room temperature (RT) is formed by the coexistence of trans and gauche isomers. The trans-isomer increases the defects in the lattice and thus decreases the activation energy for ion migration. Also in trans-gauche isomeric, which includes molecules rotating around the central C–C bond, the SN molecule contributes to increasing the ion mobility [43]. Yet, the mechanical strength of such solid-state electrolytes is not sufficient for their practical applications. Therefore, the incorporation of high-strength polymers is the main way to solve the problem. Zhou et al. [44] prepared a solid-state electrolyte based on nitrile material. Cyanoethyl polyvinyl alcohol (PVA-CN) was polymerized in situ in the SN-based solid-state electrolyte. This solid-state electrolyte was filled in a PAN fiber network. The cross-linked PVA-CN polymer backbone enhances the mechanical strength of the SN. PVA-CN/SN SPEs exhibit appreciable ionic conductivity of 0.3 S cm^−1^.

**Table 1:** The molecular weight of common polymers

Polymer	Mn (g mol^−1^)	References
PEO	≤ 10^3^	[45, 46]
	10^3^–10^5^	
	≥ 10^6^	
PEGDA	10^2^–10^4^	[47, 48]
PPC	≤ 10^5^	[49–51]
PTMC	≤ 10^6^	[52]
PMMA	≤ 10^4^	[53]
PEGDMA	≤ 10^3^	[54, 55]
PVC	≤ 10^6^	[56–58]
PAN	≤ 10^6^	[59, 60]
PVDF	10^5^–10^6^	[61, 62]
PVDF-HFP	10^5^–10^6^	[63, 64]

Currently, the plastic crystal materials used for SPEs are mostly nitrile materials. However, the compatibility between nitrile and lithium metal is poor. As well, the mechanical strength of nitriles is low. Modified lithium metal, with a supporting membrane, mixed with high-strength polymer can solve the above problems. The research on plastic crystal materials is still in the beginning stage, and more research is needed to succeed.

### Inorganic Fillers

The uniform mixing of inorganic fillers with polymers has been extensively investigated. Inorganic fillers in polymers reduce the tendency of the polymer to crystallize and accelerate the lithium salt dissociation. Furthermore, such an abundant composite solid electrolyte interface may provide multiple transfer routes for lithium ions, resulting in improved ionic conductivity. Inorganic fillers can be grouped into two categories: passive fillers and active fillers.

#### Passive Fillers

Passive fillers are lithium-ion insulators. They cannot conduct lithium ions by themselves. However, the existence of these fillers can affect the ability of polymer chain segments to transport ions [65]. First, passive filler is added to the polymer matrix as small molecule plasticizers. This can increase the amorphous phase in the polymer matrix, thus inhibiting the polymer crystallization kinetics and reducing the Tg. Moreover, with an increase in the localized amorphous region, the ion transport efficiency is elevated. Second, based on Lewis acid–base theory, the surface groups of passive fillers would interact with ion pairs to promote further dissociation. In recent decades, many passive fillers, including TiO_2_ [66], Al_2_O_3_ [67], SiO_2_ [68] and ZrO_2_ [69]_,_ have been widely applied in CPEs owing to their advantages of easy synthesis, controllable size and stable physical and chemical stability. Table 2 shows typical passive fillers and their ionic conductivity. There is another type of passive filler called ferroelectric ceramic fillers, such as BaTiO_3_ [70]. Different from oxide fillers, ferroelectric ceramic fillers interact with polymer chains through spontaneous polarization to improve the ionic conductivity in the interfacial region. Besides, clays are also involved. This kind of passive filler can provide a large specific surface area. The free lithium ions are increased at the interfacial area between polymers and fillers. However, the mechanism of this interaction is relatively complex, and there is no clear explanation for this process.

**Table 2:** CPEs incorporated with passive fillers

Passive fillers	Polymer	Ionic conductivity (S cm^−1^)	Temperature (°C)	References
TiO_2_	PPC	1.52 × 10^−4^	RT	[71]
SiO_2_	PPC	8.5 × 10^−4^	60	[72]
SiO_2_	PEO-PEGDA	1.1 × 10^−4^	30	[73]
Mg_2_B_2_O_5_	PEO	1.53 × 10^–4^	40	[74]
V_2_O_5_	PVDF	2.2 × 10^−3^	RT	[75]
UIO-66@67	PEO	9.2 × 10^−4^	25	[76]
CeO_2_	PEO	1.1 × 10^−3^	60	[77]
MnO_2_	PEO	1.95 × 10^−5^	30	[78]
ZIF-8	PEO	2.2 × 10^−5^	30	[79]
ZrO_2_	PMMA-SAN	2.32 × 10^–4^	RT	[80]
ZnO_2_	PEO	1.5 × 10^−5^	25	[81]
Ce-MOF	PEO	3.0 × 10^−5^	30	[82]
BaTiO_3_	PEO	1.5 × 10^−5^	25	[83]
Y_2_O_3_	PEO	5.95 × 10^−5^	RT	[84]
Halloysite nanotubes	PEO	9.23 × 10^–5^	25	[85]

#### Active Fillers

Compared to passive fillers, lithium fast-ion conductors serving as active fillers can improve the electrochemical performance of CPEs more effectively by facilitating the migration of lithium ions. Table 3 shows the ionic conductivity of typical active fillers incorporated with polymers. Active fillers always exhibit a high ion conductivity (> 10^–4^ S cm^−1^). This can be attributed following factors: The many continuous defects in active fillers with low activation energy enable easy ion hopping. Moreover, active fillers themselves can supply a large number of lithium ions, enhancing the concentration of free lithium ions at the interface between the active filler and the polymer. Therefore, the total ionic conductivity is improved. Generally, active fillers include perovskite, garnet, LISICON, etc. When the percentage of active filler is less than 40 wt%, the CPEs can supply a high concentration of free lithium ions. However, the concentration of active filler exceeds a certain threshold, it forms a fully permeable network. At this moment, the ion transport behavior changes.

**Table 3:** CPEs incorporated with active fillers

Active fillers	Polymer	Ionic conductivity (S cm^−1^)	Temperature (°C)	References
LGPS	PEO	8.01 × 10^−4^	60	[86]
LGPS	PEO	1.21 × 10^−3^	80	[87]
LLTO	PEO	0.16 × 10^−3^	24	[88]
LLTO	PAN-PVDF	1.43 × 10^−3^	RT	[89]
LLTO	PVDF	2.37 × 10^−3^	RT	[90]
LLZTO	PEO	3.03 × 10^−4^	55	[91]
Li_3_PS_4_	PEO	8.4 × 10^−6^	RT	[92]
LATP	PEO	1.2 × 10^−5^	60	[93]
LSZT	PVDF	6.26 × 10^−5^	20	[94]
LLZAO	PEO	1.33 × 10^−4^	25	[95]
LLZO	PEO-PVDF	4.2 × 10^−5^	30	[96]
LAGP	PEO	6.76 × 10^− 4^	60	[97]

### Distribution of Fillers in Polymers

The incorporation of inorganic fillers with polymers can allow one to take full advantage of CPEs. For example, inorganic fillers can be used to elevate the ionic conductivity, t_Li_^+^ and electrochemical stability window of SPEs [98]. Besides, they also show excellent performance in alleviating the interfacial stability between the electrolyte and electrode. Therefore, in recent years, CPE has a broad application prospect in the field of lithium batteries and has attracted more and more attention.

In the early phases of research, scholars were devoted to the Lewis acid–base interactions between inorganic fillers and polymers. This model assumes that fast-ion-conducting channels can be constructed on the surface of fillers. Since then, many studies have focused on the construction of fast-ion-conducting channels. This fast-ion transfer percolation channel is related to the orientation (ordered or disordered arrangement) and morphology (1D, 2D, 3D) of the filler in the polymer. Therefore, the main goal of this section is to present the integration method of inorganic fillers in CPEs and their influence on the ionic conductivity.

#### Disordered Fillers in CPEs

Usually, inorganic fillers are mainly dispersed disorderly in the polymers. The presence of inorganic fillers disturbs the crystallization of the polymers and thus increases the ionic conductivity of CPE. However, the fillers inevitably prefer to aggregate in the polymer, which hinders the formation of percolation network. Facilitating the dispersion of fillers in polymers is an effective method for forming percolation networks [99].

**Fig. 2:**
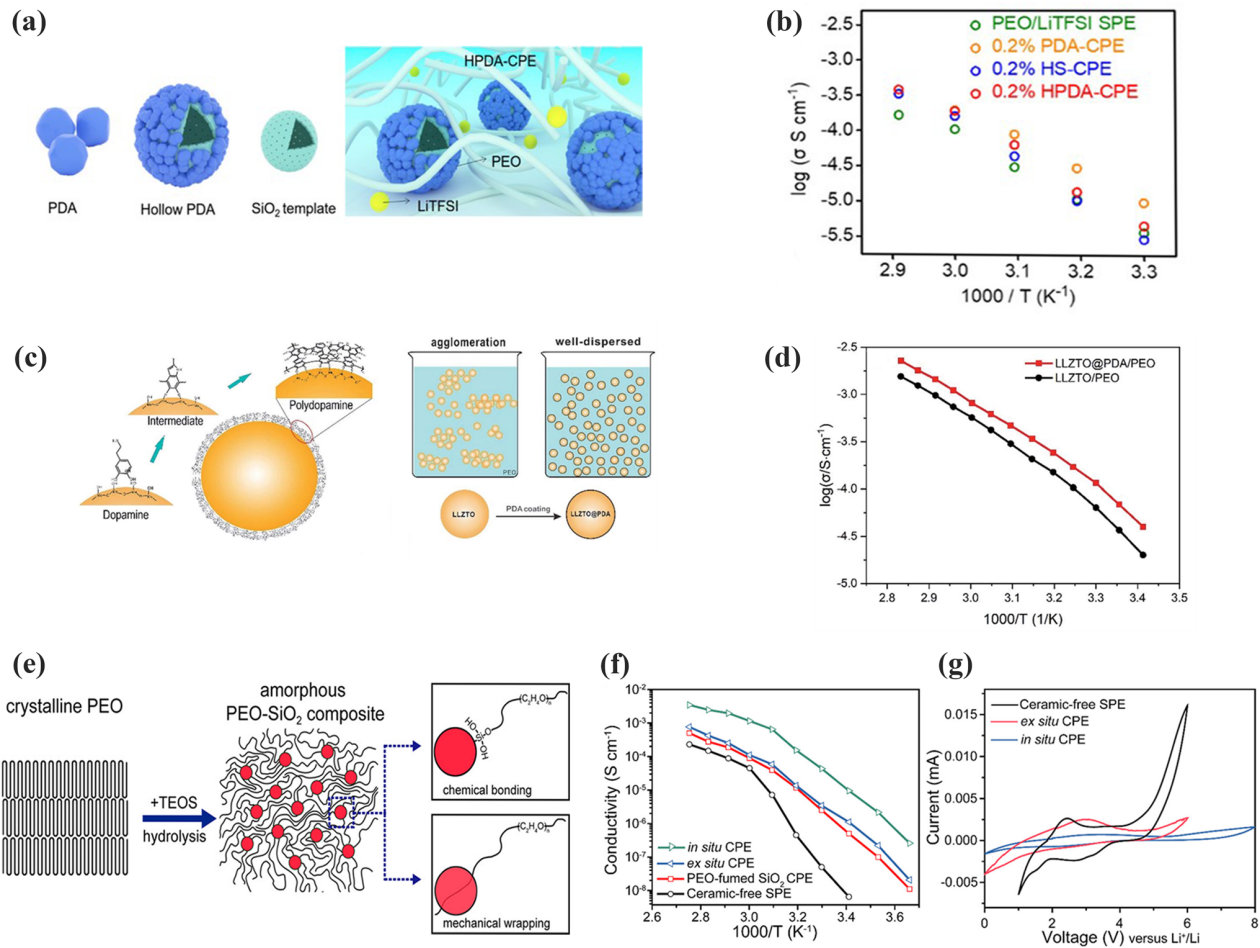
**a** Schematic of HPDA-PEOCPEs; **b** Arrhenius plots for HPDA-PEO CPEs. Adapted with permission from Ref. [100]. **c** Schematic of dopamine on the surface of LLZTO particles; **d** Arrhenius log $\sigma \sim$ 1000/*T* of LLZTO@PDA-PEO and LLZTO/PEO CPEs. Adapted with permission from Ref. [101]. **e** Schematic diagram illustrating the in situ hydrolysis process and the interaction mechanism between PEO chains and SiO_2_; **f** Arrhenius plots of SiO_2_-PEO CPEs; **g** Electrochemical stability windows of SiO_2_-PEO CPEs. Adapted with permission from Ref. [102]

Li et al. [100] prepared HPDA fillers, as shown in Fig. 2a, in which hollow silica was used as a template and covered with a layer of polydopamine. Compared with silica alone, the thin polydopamine layer facilitated the dispersion of HPDA in PEO by providing a surface that was more compatible with the PEO matrix. As a consequence, the ionic conductivity of HPDA-PEO CPEs was 0.189 × 10^−3^ S cm^−1^ (60 °C), as shown in Fig. 2b. Huang et al. [101] coated a layer of polydopamine (PDA) in situ on the surface of LLZTO. The modified LLZTO with PDA allowed uniform dispersion of LLZTO (80 wt%) in SPEs. PDA lowed the surface energy of LLZTO to promote the dispersion of LLZTO nanoparticles in the polymers (Fig. 2c). Thus, the ionic conductivity of LLZTO@PDA-PEO CPEs was increased to 1.1 × 10^–4^ S cm^−1^ (at 30 °C) (Fig. 2d). Cui and workers [102] introduced a method for the in situ production of inorganic fillers in SPEs (Fig. 2e). Thanks to this in situ polymerization, SiO_2_ formed a continuous dispersed phase in the polymer. Thus, more contact area was provided for Lewis acid–base interactions. Moreover, the mono-dispersity SiO_2_ effectively inhibited the crystallization of PEO to promote the movement of polymer segments. As a consequence, the SiO_2_-PEO CPEs showed a superior ionic conductivity of 4.4 × 10^–5^ S cm^−1^ at 30 °C (Fig. 2f). Moreover, the electrochemical window was broadened to 5.5 V versus Li/Li^+^ (Fig. 2g).

**Fig. 3:**
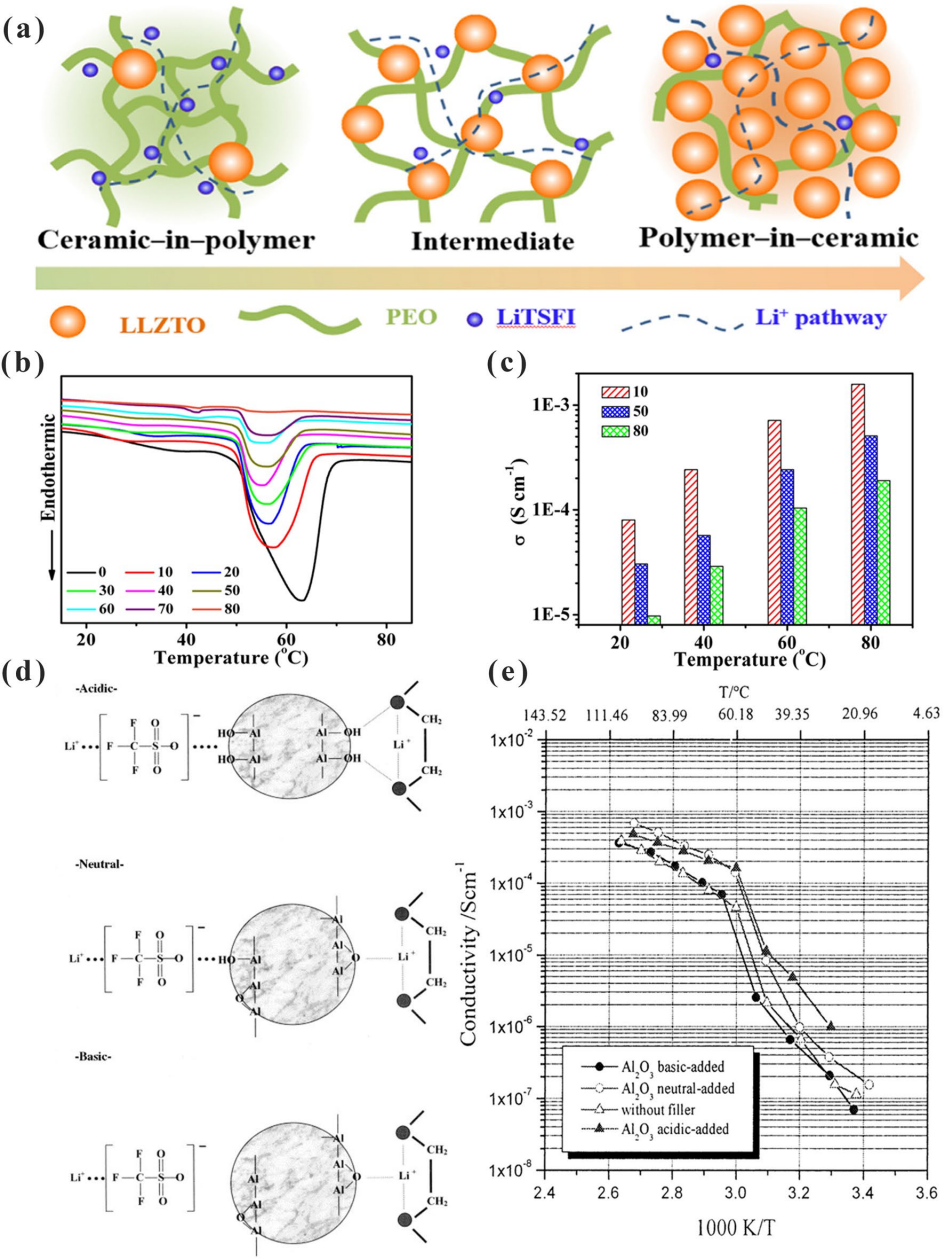
**a** Schematic illustration for LLZTO- PEO CPEs; **b** DSC result of different filler contents of LLZTO-PEO CPEs; **c** Ionic conductivities of different filler contents of LLZTO- PEO CPEs. Adapted with permission from Ref. [103]. **d** Surface interactions between three different type Al_2_O_3_ and PEO; **e** conductivity plots of Al_2_O_3_-PEO CPEs. Adapted with permission from Ref. [104]

Chen et al. [103] prepared LLZTO-PEO CPEs by the hot-pressing technique. The CPEs, including fillers incorporated into polymers and polymers incorporated into fillers, were designed by adjusting the content of LLZTO (Fig. 3a). As illustrated in Fig. 3b, Tm of LLZTO- PEO CPEs decreases gradually with the addition of LLZTO particles. When the LLZTO concentration was low enough, the fillers were well dispersed in the polymers causing less crystallization of the polymers. However, when the LLZTO content exceeded the permeation threshold, it could not be dispersed uniformly, which caused a significant increase in the stiffness of LLZTO-PEO CPEs. With the increase in LLZTO content, the ionic conductivity first increased and then decreased, which is due to the serious agglomeration of the additional LLZTO. Figure 3c shows that the ionic conductivity of LLZTO-PEO CPEs got a maximum value of 1.17 × 10^–4^ S cm^−1^ at 10% LLZTO. Croce et al. [104] investigated the mechanism of ionic conductivity enhancement for Al_2_O_3_ with different surface treatments in PEO. As shown in Fig. 3d, there were three different surface interactions between Al_2_O_3_ and PEO. It was assumed that a Lewis acid (Li^+^) interacted with a Lewis base (–OH groups of Al_2_O_3_). The additional interactions weakened the complexation of lithium ions with oxygen atoms on the PEO chain to facilitate the transport of lithium ions. As shown in Fig. 3e, the differences in ionic conductivity were directly related to the different filler surfaces. This can be ascribed to the different microstructural interactions that occurred when varying the type of ceramic surface states. We will discuss this interaction in detail in the next section.

**Fig. 4:**
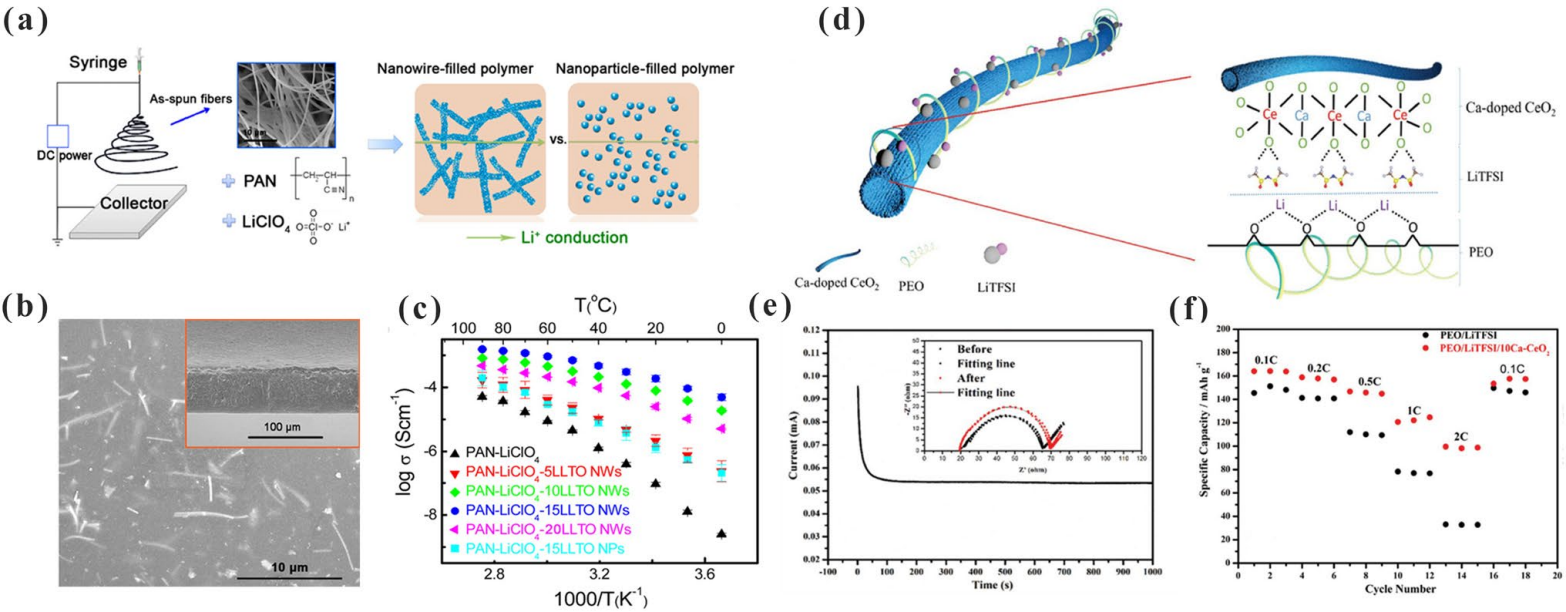
**a** Lithium-ion pathways in nanowire- and nanoparticle-filled PAN CPEs; **b** SEM pictures for the PAN-LLTO NWs; **c** Arrhenius plots of the PAN-LLTO NWs and PAN-LLTO NPs CPEs. Adapted with permission from Ref. [105]. **d** Diagram of the enhanced mechanism of lithium-ion transport in Ca–CeO_2_-PEO CPEs; **e** Chronoamperometry curves of Ca–CeO_2_-PEO CPEs; **f** Rate performance of PEO-LiTFSI and Ca–CeO_2_-PEO CPEs with LiFePO_4_ cathode. Adapted with permission from Ref. [106]

In addition to the above-mentioned 0D inorganic fillers, which are randomly dispersed, there are some 1D inorganic fillers that are also randomly dispersed in the polymers. Liu et al. [105] first fabricated LLTO nanowires by electrostatic spinning and dispersed them in PAN to prepare PAN- LLTO NW CPEs (Fig. 4a). LLTO nanowires with a high length-to-diameter ratio can provide continuous transport channels for lithium ions. Furthermore, they can be uniformly distributed in the polymer matrix as indicated in Fig. 4b. As shown in Fig. 4c, the ionic conductivity of PAN-15LLTO NW CPEs was higher (2.4 × 10^–4^ S cm^−1^) than that of PAN-15LLTO NP CPEs. Subsequently, Chen et al. [106] added Ca–CeO_2_ nanotubes into PEO to prepare Ca–CeO_2_–PEO CPEs. Ca–CeO_2_ nanotubes can inhibit the reorganization and increase the dipole moment of PEO chains. As depicted in Fig. 4d, Ca–CeO_2_ nanotubes can accelerate the dissociation of LiTFSI through oxygen vacancies on the surface, resulting in more free lithium ions. The Ca–CeO_2_–PEO CPEs offered a high t_Li_^+^ of 0.453 (Fig. 4e). Moreover, the Li|Ca–CeO_2_–PEO CPEs|LiFePO_4_ battery provided an initial discharge capacity of 164 mAh g^−1^ at 0.1C. Even at a high current density of 2C, 100 mAh g^−1^ was obtained (Fig. 4f). After 200 cycles, the discharge capacity was maintained at 93 mAh g^−1^ at 1C.

In addition, 2D fillers are also of great interest due to their structural characteristics. In practical applications, small-sized 2D nanosheets are more popular among researchers. This is due to the fact that large sizes of 2D nanosheets are difficult to provide continuous ion transport paths. And, the larger size 2D nanosheets offer limited ability to inhibit the crystallization of polymeric matrix. However, 2D fillers are equipped with high specific surface area, ultrathin lamellar structure and large aspect ratio. Once the size of the 2D nanosheet is small enough, a larger contact area can be formed between it and the polymer matrix. A new ionic conductivity will be established between the 2D nanosheet–polymer interfaces, resulting in a higher ionic conductivity. Shi et al. [107] prepared an MXene-based silica nanosheet MXene-mSiO_2_. Due to the large specific surface area of MXene-mSiO_2_ and the abundance of functional groups on the surface, a large number of Lewis acid–base interactions existed in the MXene-mSiO_2_-PPO interface. These interactions promote the rapid conduction of lithium ions. MXene-mSiO_2_-PPO CPEs provide an ionic conductivity of 4.6 × 10^–4^ S cm^−1^. Rojaee et al. [108] prepared BP-PEO CPEs using a new 2D material, black phosphorus (BP). The unique curved structure of BP nanosheets allows the ions to be anisotropic at the interface. BP nanosheets can effectively trap TFSI- as well as weaken the bond length of N–Li. Therefore, the dissociation of Li^+^ is promoted. And Li/BP-PEO CPEs/Li cells can be cycled for more than 500 h at RT. Besides, graphene, vermiculite and double hydroxide also have a flake structure. Luo et al. [109] reported an ultrathin vermiculite nanosheet VS. The VS-PEO CPEs could provide ionic conductivity of 1.2 × 10^–3^ S cm^−1^. In addition, the excellent mechanical strength and enhanced dimensions stability of VS-PEO CPEs were favorable to inhibiting the growth of lithium dendrites.

#### Ordered Fillers in CPEs

The above-mentioned nanoparticles or nanowires tend to be randomly dispersed in the polymer matrix. This structure is thermodynamically stable, which makes it difficult for the fillers to form a continuous conduction route. The ion-conducting pathways constructed by randomly dispersed microstructures are undesirable. To obtain more efficient ion transport, researchers have focused on CPEs that are prepared with directionally aligned ceramic fillers.

**Fig. 5:**
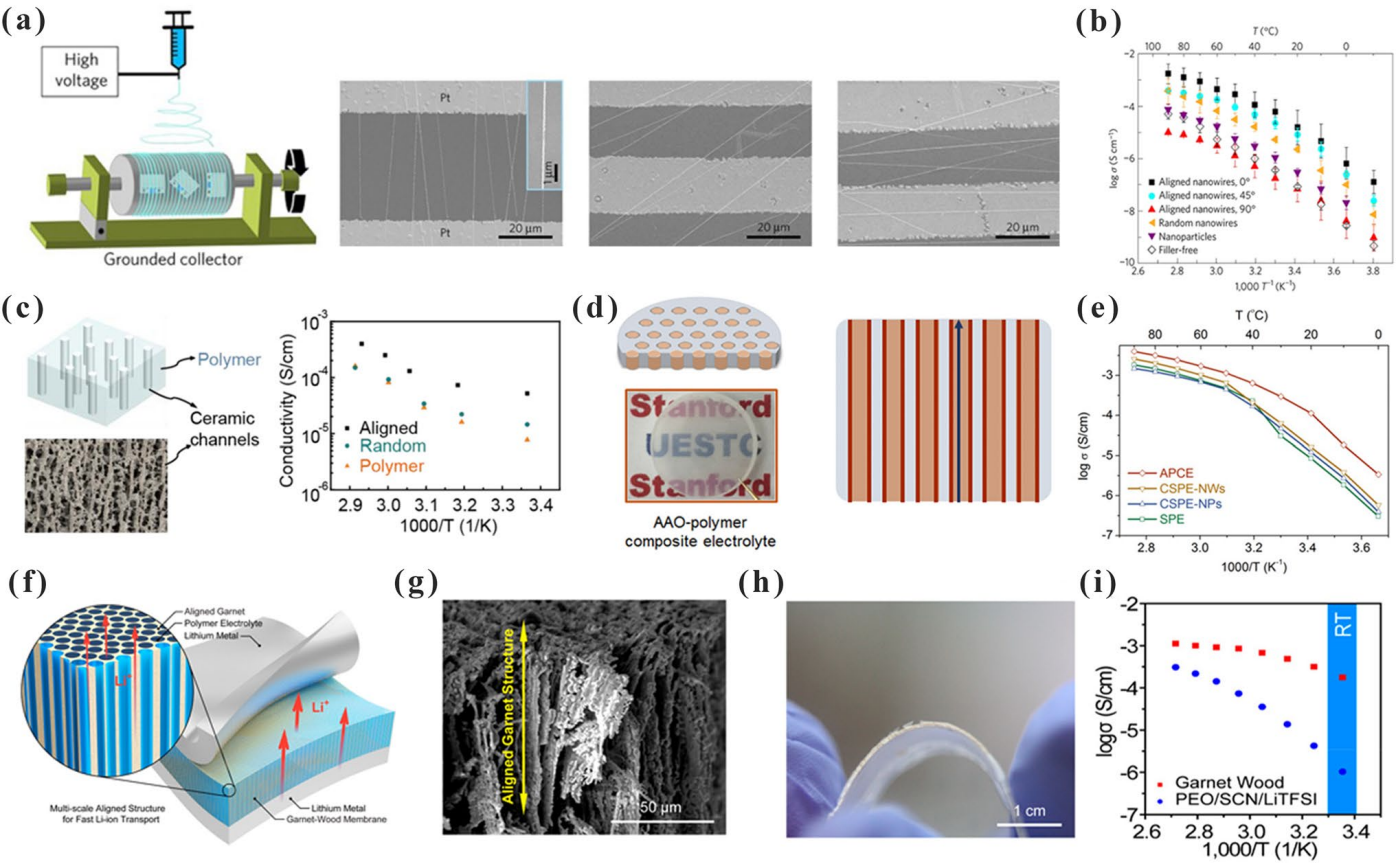
**a** CPEs with different aligned LLTO nanowires; **b** Arrhenius plots of different aligned LLTO-PAN CPEs. Adapted with permission from Ref. [110]. **c** Schematic diagram of vertically aligned LATP in polymers and the ionic conductivity plots. Adapted with permission from Ref. [111]. **d** Schematics of AAO-PEO CPEs; **e** Interfacial ionic conductivities of CPEs based on AAO disks. Adapted with permission from Ref. [112]. **f** Schematic of multiscale aligned LLZO incorporated with PEO; **g** SEM images showing the alignment of channels of LLZO-PEO CPEs; **h** Photograph of the LLZO-PEO CPEs; **i** Ionic conductivity of LLTO-PEO CPEs and PEO SPEs. Adapted with permission from Ref. [113]

Liu et al. [110] investigated the influence of LLTO nanowires of different orientations on lithium-ion transport. As shown in Fig. 5a, LLTO nanowires with different orientations (angles of 0° (perpendicular), 45° and 90° (parallel)) were prepared by adjusting different positions of the collector. The ionic conductivities of the LLTO-PAN CPEs made by randomly LLTO nanowires and orientation-ordered LLTO nanowires (angles of 0°, 90° and 45°) were 7.82 × 10^–6^, 5.02 × 10^–5^, 1.78 × 10^–7^ and 2.24 × 10^–5^ S cm^−1^ at 30 °C, respectively (Fig. 5b). The randomly dispersed LLTO nanowires formed a semicontinuous structure in CPEs, which facilitated the transportation of lithium ions. However, the ionic conductivity of CPEs further increased when the orientation was parallel to the current direction, forming a continuous fast-ion transport channel. Thereafter, Zhai et al. [111] added a continuous vertical arrangement of Li_1+x_Al_x_Ti_2−x_(PO_4_)_3_ (LATP) in PEO/PEG (Fig. 5c). The vertically aligned LATPs formed an efficient ionic conductivity structure with an excellent ionic conductivity of 5.2 × 10^–5^ S cm^−1^ at RT. This value is approximately 3.6 times higher than that of LATP NP-PEO/PEG CPEs (1.5 × 10^–5^ S cm^−1^). Zhang et al. [112] also reported CPEs with a vertically continuous structure. As shown in Fig. 5d, surface-modified anodic aluminum oxide (AAO) acted as a ceramic backbone rich in continuous nanoscale channels. PEO was packed in the pore channel. These AAO-PEO CPEs allowed fast lithium-ion transport along the AAO-PEO interface. The ionic conductivity of the AAO-PEO CPEs was 5.82 × 10^–4^ S cm^−1^ (Fig. 5e). Dai et al. [113] exploited highly conductive garnet frameworks equipped with multiscale aligned structures through a top-down method. PEO was doped into the vertically aligned garnet nanostructure to produce flexible LLZO-PEO CPEs (Fig. 5f). The LLTO framework inherited the aligned porous structure of the wood template (Fig. 5g). Moreover, the LLTO-PEO CPEs were flexible (Fig. 5h). They possessed an excellent ionic conductivity of 1.8 × 10^–4^ S cm^−1^ at RT (Fig. 5i).

#### Three‐Dimensional (3D) Fillers in CPEs

The filler is easily clustered in the polymer matrix. The construction of a 3D skeleton structure by controlling the space position of the filler in the polymer is an effective way to solve this dispersion problem. Moreover, the inorganic network has high mechanical strength, which can hinder lithium dendrite growth and promote cyclic stability performance.

**Fig. 6:**
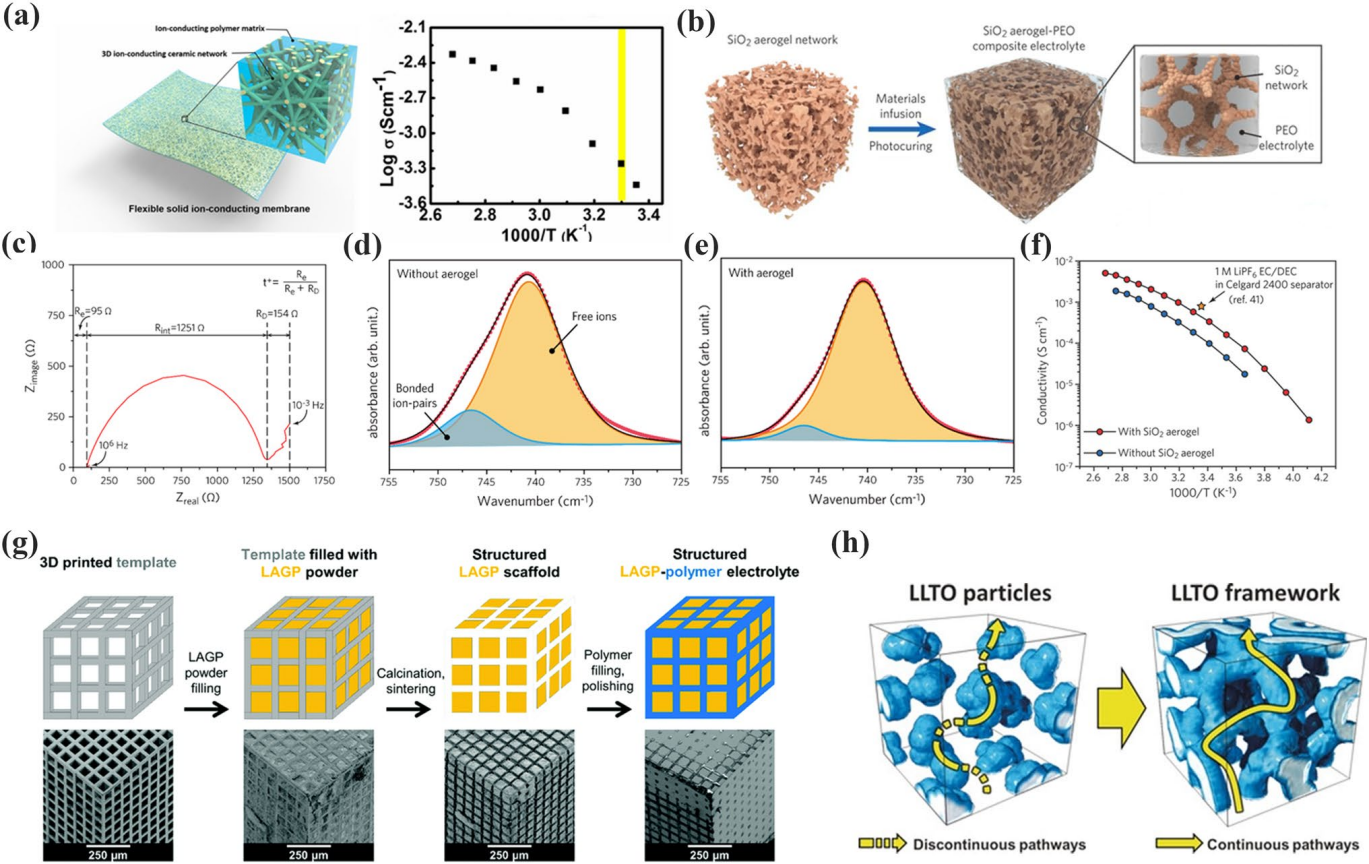
**a** Schematic and ionic conductivity of the LLZO-PEO CPEs. Adapted with permission from Ref. [114]. **b** Schematic of the SiO_2_-aerogel-reinforced CPE; **c** Nyquist plot of electrochemical impedance spectroscopy of Li|SiO_2_-PEO CPEs|Li cell; **d–e** FTIR spectra of the electrolytes without and SiO_2_ aerogel; **f** ionic conductivity plot of CPEs with and without SiO_2_ aerogel. Adapted with permission from Ref. [115]. **g** A diagram of the temples used for the LAGP-PEO CPEs and SEM images. Adapted with permission from Ref. [116]. **h** Agglomerated nanoparticles and three-dimensional continuous framework of LLTO. Adapted with permission from Ref. [117]

Fu et al. [114] prepared LLZO-PEO CPEs consisting of interconnected LLZO nanowires and PEO. The three-dimensional interconnected LLZO nanowires effectively precluded the agglomeration of nanoparticles and formed a continuous lithium-ion conduction network, as depicted in Fig. 6a. The ionic conductivity of the LLZO-PEO CPEs was 2.5 × 10^–4^ S cm^−1^ at RT. The SiO_2_ 3D network structure-enhanced CPEs were fabricated by in situ hydrolysis by Cui et al. [115]. As shown in Fig. 6b, the 3D structure of SiO_2_ has a high specific surface area (701 m^2^ g^−1^) and continuous ion transport channels. This special 3D structure enhanced the Lewis interactions and boosted the t_Li_^+^ of SiO_2_-PEO CPEs (t_Li_^+^ = 0.38) (Fig. 6c). The strong Lewis acid–base interactions promote the separation of anions and cations. As shown in Fig. 6d–e, the dissociation of LiTFSI in SiO_2_-PEO CPEs increased from 84.7 to 94.4%. The ionic conductivity of SiO_2_-PEO CPEs was 0.6 × 10^–3^ S cm^−1^ at 30 °C (Fig. 6f). The Li|SiO_2_-PEO CPEs|LFP cell exhibited a good performance (105 mAh g^−1^ at 0.4C), even at 15 °C. It is clear that facilitating continuous ion conduction pathways is a good strategy for promoting lithium-ion migration.

Bruce et al. [116] designed gyroscopically structured CPEs by 3D printing (Fig. 6g). This structure formed a bi-continuous ion conduction pathway, in which the LAGP ceramic backbone ensured fast Li-ion transport and the polymers guaranteed the efficient dissociation of lithium ions and the flexibility of CPEs. This structure exhibited a promising ionic conductivity of 1.6 × 10^–4^ S cm^−1^ at RT. Bae et al. [117] fabricated a 3D LLTO framework for high-performance CPEs. Figure 6h shows the 3D structure of LLTO with a high content ceramic (44 wt%). In addition, the ionic conductivity was increased to 8.8 × 10^–5^ S cm^−1^ at RT.

In summary, although disordered nanoparticles can reduce the crystallinity of PEO and promote the conduction of lithium ions through Lewis acid–base interactions, the discontinuous lithium-ion transport path and the tendency of nanoparticles to agglomerate lead to worse ion conduction. In contrast, some ordered structures, especially 1D nanowires aligned parallel to the lithium-ion transport direction, can provide the shortest lithium-ion transport paths. Therefore, the smooth ion conduction in 3D continuous structures is the main direction for future development.

### Filler–Polymer Interface

As mentioned above, the ionic conductivity of CPEs can be significantly increased by inorganic fillers doped in polymers. This is due to the Lewis acid–base interaction in filler–lithium salt–polymer. Significantly, Lewis acid–base interactions promote further dissociation of the lithium salt and increase the free Li^+^ concentration in the polymer. Moreover, that Lewis acid–base interaction is much more obvious in the interfacial phase of the filler–polymer. This is highly related to the type, size, concentration, morphology and surface properties of the inorganic fillers. Constructing fast-ion conduction channels at the filler–polymer interface is an effective way to enhance the ion transport efficiency.

In order to enhance the ion transport efficiency at the filler–polymer interface, Cheol et al. [118] used purine-modified MOFs as inorganic fillers to enhance CPEs. First, strong hydrogen bonds exist between –NH_2_ on the surface of Bio-MOF11, which promotes the dispersion of Bio-MOF11 in PEO and facilitates to increase the ion transport-specific surface area. Secondly, the open metal sites (Lewis acidic) can effectively trap the anions by electrostatic interaction. Therefore, the multiple Lewis basic/acidic sites in the Bio-MOF11-PEO CPEs effectively enhance the lithium-ion transport efficiency. Zhou et al. [119] prepared a novel amphoteric ion-modified metal–organic framework NH_3_^+^–SO_3_^−^@ZIFs. At the interface of PEO– NH_3_^+^–SO_3_^−^@ZIFs, the strong electrostatic interaction between the cation and TFSI^−^ largely inhibited the movement of the anion and enhanced the t_Li_^+^. Chen et al. [120] coated a layer of PDA on the surface of Co_3_O_4_. The PDA coating can act as a multifunctional medium to finely adjust the ion distribution and transport behavior through Lewis acid–base interactions. The phenolic hydroxyl and o-benzoquinone groups on the surface of the Co_3_O_4_@PDA not only alleviate the coordination of PEO with Li^+^, but also the –NH^−^ can form hydrogen bonding network with PEO chains. This can increase the amorphous region of PEO and form an effective ion migration pathway at the Co_3_O_4_@PDA-PEO surface to improve the ionic conductivity.

Apart from the special interactions between the filler–polymer which affect the formation of the ion permeation network, the size and concentration of the inorganic fillers also have a great influence on the properties of the filler–polymer interface. To increase the contact area of filler–polymer, some fillers with smaller particle size and larger specific surface area are often used. Hu et al. [121] compared the effects of different sizes of ZrO_2_ (220, 365, and 470 nm in diameter, respectively) on the formation of ion permeation networks in PAN-LiClO_4_. The results showed that the ionic conductivity of ZrO_2_-PAN CPEs increased with the decrease in the size of ZrO_2_. In comparison, ZrO_2_ (220 nm) can form more effective ion transport interfaces. So, ZrO_2_ (220 nm)-PAN CPEs have the best ionic conductivity of 1.16 × 10^–3^ S cm^−1^. This is for the passive inorganic fillers. At the same time, the filler size has a similar effect on the active filler. For example, Zhang et al. [122] investigated the effect of the active fillers of LLZTO with different sizes (10 um, 400 nm, 40 nm) on ionic conductivity. Excluding the disturbance of lithium salts in CPEs, LLZTO (40 nm)-PEO CPEs exhibited a greater ionic conductivity than LLZTO (10 um)-PEO CPEs. The enhanced ionic conductivity of the smaller LLZTO is attributed to the remarkably high conductive routes along the interface of PEO-LLZTO. And the small particles usually have a relatively large specific surface area, leading to an increase in the coherent conductivity path.

When the size of the filler is certain, the variations in the concentration of the filler also greatly influence the ion transport behavior in CPEs. With a small volume of passive filler in CPEs, the fast ionic conductivity region at the filler–polymer interface increases with the increase in filler. At this time, the ionic conductivity will show the same tendency. However, with the increase in passive filler, especially some nano-sized inert fillers, it tends to agglomerate. The unfavorable dispersion will reduce the filler–polymer contact area and cause a negative growth in ion transport rates. Xu et al. [123] prepared Bi/HMT-MOFs-PEO CPEs. It was found that the ionic conductivity of Bi/HMT-MOFs-PEO CPEs showed a phenomenon of increasing first and then decreasing. When Bi/HMT-MOFs were increased to 10 wt%, Bi/HMT-MOFs-PEO CPEs exhibited the highest ionic conductivity (3.06 × 10^–5^ S cm^−1^, 25 °C). The excess amount of Bi/HMT-MOFs may lead to difficulty in forming continuous lithium-ion transport channels, and thus the ionic conductivity decreases when the filler content exceeds 10 wt%. However, these changes in the active filler are different from the passive filler. At first, the active filler does not create a continuous interfacial phase with the polymer phase, in which ionic transport does not occur in the bulk phase of the active filler. Therefore, the ionic conductivity tends to first increase and then decrease with a change in active filler concentration. But, as the concentration of active filler continues to increase, the new ion pathways will be established inside the CPEs. Therefore, the ionic conductivity will continue to increase again. Wang et al. [124] systematically investigated the influence of LATP content on ion permeation channels. The results indicated that at low content, LATP (4 vol%)-PEO CPEs exhibited a high ionic conductivity of 1.70 × 10^–4^ S cm^−1^. The obvious enhancement of ionic conductivity was attributed to the rapid migration of lithium ions within the LATP-PEO surface. As the LATP increases, the ionic conductivity of LATP-PEO CPEs starts to decrease. However, when the LATP increases to 13 vol%, the volume fraction of the interfacial phase can reach a maximum. At this moment, it was derived that the ionic conductivity of (13 vol%)-PEO CPEs was showing an increasing trend again.

## Effects of Fillers and the Mechanism in CPEs

**Fig. 7:**
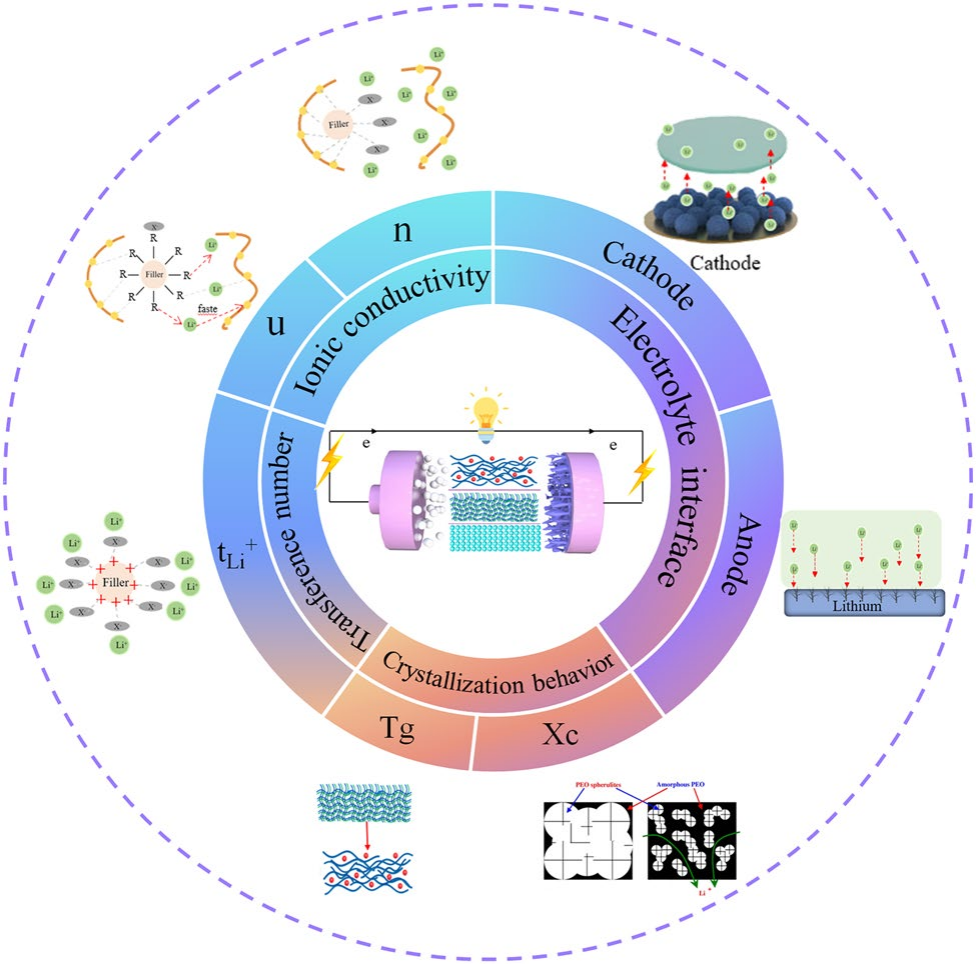
Schematic of the effects of fillers in CPEs for lithium batteries

CPEs consist of polymer matrix, lithium salt and inorganic filler. In general, SPEs are strongly limited in terms of ionic conductivity by the high crystallinity. Fortunately, CPEs prepared by introducing fillers in SPEs can effectively suppress the crystallization behavior of polymers, which is indicated to be a more promising method for the development of SSLBs [12]. Inorganic fillers can promote the comprehensive electrochemical performance of CPEs, but this mechanism is complex and involves many significant factors such as ionic conductivity, t_Li_^+^, and polymer aggregate structure [11]. The complex relationship is shown in Fig. 7.

In CPEs, the polymer, inorganic filler and lithium salt interact with one another. This interaction mainly occurs in two aspects:

1. The interaction between the filler and the lithium salt. This involves the alteration of the lithium-ion chemical environment. And reflected mostly in the changes in ionic conductivity and t_Li_^+^.
2. The interaction between the filler and the polymer. This involves changes in the polymer aggregate structure. It can be characterized by the Xc, Tg and spherulites.

In addition to the above two main aspects, some functionalized fillers simultaneously interact with lithium salts and polymers to change the coordination mode between polymers and lithium ions, which is also worthy of further consideration. In the following sections, we will discuss the electrochemical enhancement mechanism of inorganic fillers for CPEs from the two main factors.

### Interactions Between Fillers and Lithium Salts

The surfaces of inorganic fillers are rich in chemical groups. These fillers exhibit strong Lewis acid–base interactions with the lithium salts. The categories of such interactions include hydrogen bond, hole, and dipole–dipole interactions [125]. On the one hand, the interaction between the lithium ions and fillers could expedite the transportation as well as enhance the ionic conductivity. On the other hand, the filler interactions with anions (TFSI^−^, ClO_4_^−^, PF_6_^−^, etc.) can enhance t_Li_^+^.

#### Ionic Conductivity

Ionic conductivity is one of the standards to measure the ionic conduction of electrolyte and a key factor in determining the electrochemical performance of SSLBs. SPEs exhibit a low ionic conductivity, which is usually in 10^–6^–10^–5^ S cm^−1^ or even much lower at RT. However, in practical applications, the ionic conductivity of solid-state electrolytes is expected to be 10^–4^ S cm^−1^. It is obvious that SPEs cannot meet the requirements. Notably, CPEs are expected to satisfy the requirements by improving the ion transport capacity.

The ionic conductivity of CPEs is given by Eq. 1 [126]:

1 $$\sigma =\sum nq \mu$$

Here, *n* is the number of carriers, *q* is the ionic charge, and *u* is the carrier mobility. For a given system, *q* is definite. Therefore, there are two pathways for boosting the ionic conductivity: (1) increase the number of carriers and (2) increase the rate of carrier motion.

1. Increase the number of carriers (*n)*

**Fig. 8:**
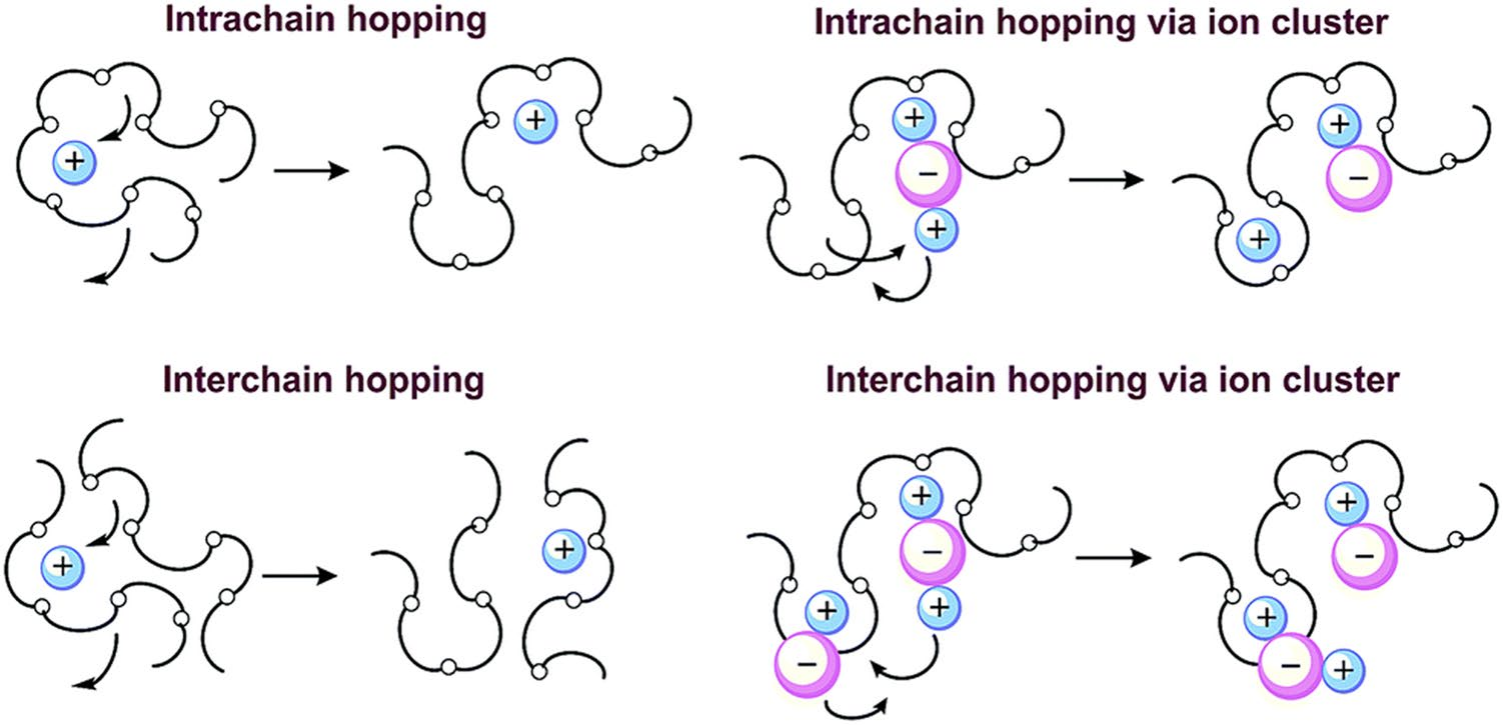
Migration of lithium ion in polymers. Adapted with permission from Ref. [127]

In CPEs, when the lithium salt concentration is sufficiently low, all the lithium ions are soluble in the polymer matrix. In these circumstances, lithium ions and anions both can act as charge carriers. However, with an increasing concentration of lithium salts, the dissolution capacity of the polymer matrix for lithium ions reaches a saturation state. As a result, electrostatic interactions between anions and cations cannot be neglected, which could reduce the number of carriers [36]. As shown in Fig. 8, lithium salt exists in the polymer in the form of ionic clusters. The migration of ionic clusters in the polymer is much more difficult. So, it is necessary to find some solutions to increase the carrier concentration.

**Fig. 9:**
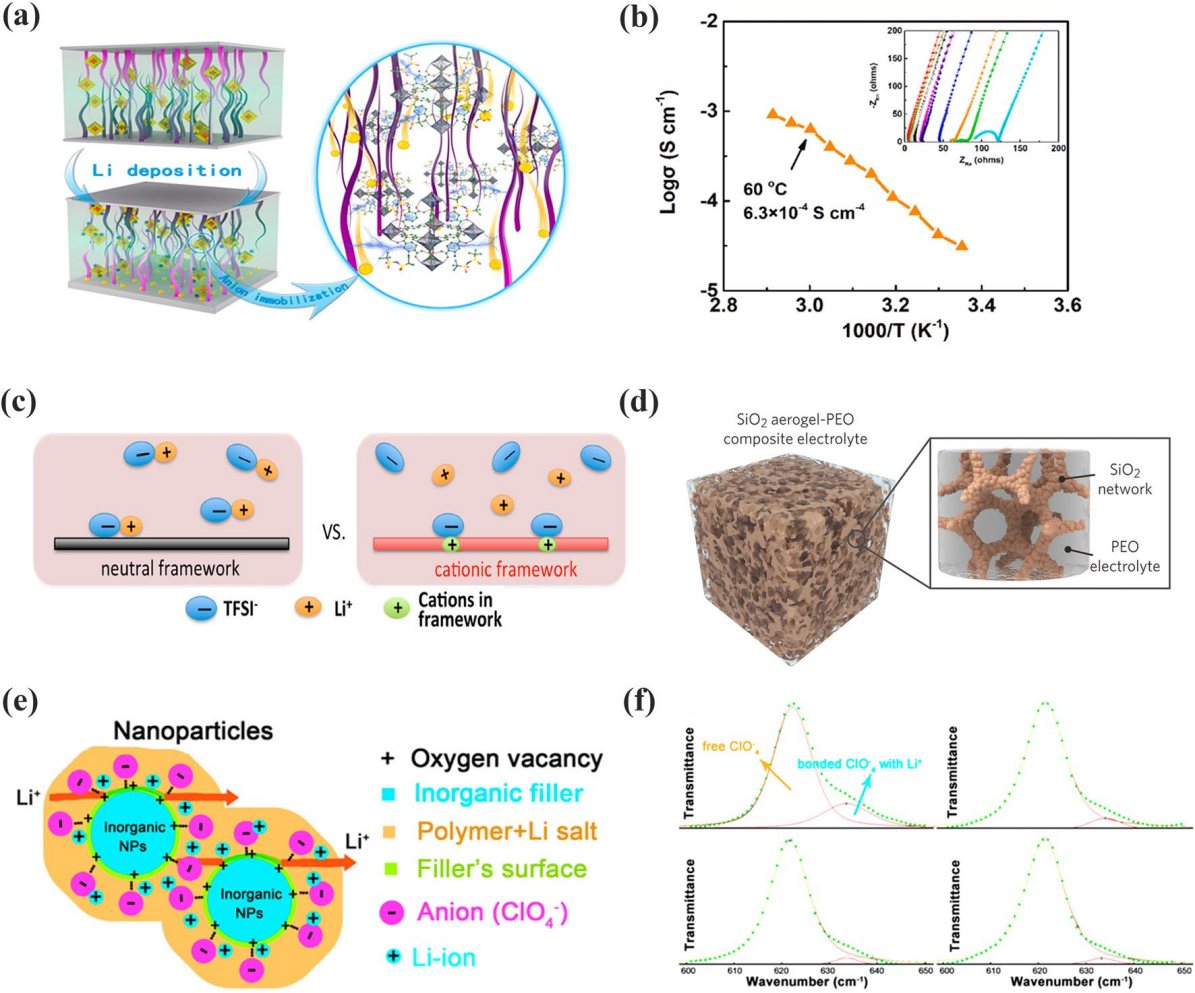
**a** Schematic diagram of Li-ion transport in CMOF; **b** the ionic conductivities of P@CMOF with different temperature. Adapted with permission from Ref. [128]. **c** Schematic illustrations of ion association in COFs with neutral and cationic frameworks, respectively. Adapted with permission from Ref. [129]. **d** LiTFSI was dissolved in PEGDA/SCN and SiO_2_ aerogel is the backbone. Adapted with permission from Ref. [115]. **e** Schematic of lithium-ion transport in YSZ; **f** FTIR spectra from filler-free electrolyte to the 2–7 mol% YSZ. Adapted with permission from Ref. [130]

Inorganic fillers incorporated with polymers are the mainstream method for increasing the ionic conductivity of CPEs. The main reason is that the inorganic filler can promote the lithium salt to dissociate, i.e., increasing the carrier concentration in the CPEs. Sun et al. [128] proposed a strategy of grafting pyridine N in UiO-66 (CMOF) (Fig. 9a). The –N^+^CH_3_ on the surface of UiO-66 interacts electrostatically with the lithium salt, which can accelerate the dissociation of the lithium salt to release a large number of carriers. As a result, the dissociation of lithium ions in the CMOF-PEO CPEs was 87.4%, which is higher than PEO-LiTFSI. This high dissociation of lithium salts endowed the CMOF-PEO CPEs with an excellent conductivity of 6.3 × 10^–4^ S cm^−1^ (at 60 °C), as demonstrated in Fig. 9b. Chen and coworkers [129] introduced cations into a COF to split the ion pairs of lithium salts by a stronger dielectric effect. As a result, the free lithium-ion concentration increased sharply at 70 °C, with ionic conductivity up to 2.09 × 10^–4^ S cm^−1^ (Fig. 9c). Cui et al. [115] doped mesoporous SiO_2_ in polymers to fabricate SiO_2_-CPEs (Fig. 9d). The interconnected SiO_2_ network had a high specific surface area and uniformly distributed pores. This maximized the interactions between SiO_2_ and lithium salts. The dissociation of LiTFSI increased from ≈ 84.7 to 94.4%. Thus, the SiO_2_-PEO CPEs displayed a high ionic conductivity of 1.0 mS cm^−1^ at 40 °C. Recently, some studies have revealed that oxygen vacancies on inorganic fillers can facilitate the decomposition of lithium salts. Liu et al. [130] reported Y_2_O_3_-doped ZrO_2_ (YSZ)-PAN CPEs, as shown in Fig. 9e. The oxygen vacancy in YSZ is positively charged and it can be used in CPEs as the Lewis acid site. As shown in Fig. 9f, the dissociation of LiClO_4_ was maximized with 7 mol% YSZ. Moreover, the conductivity of the YSZ-PAN CPEs also reached a maximum value. Zhang et al. [131] synthesized an ultrasmall Nb_2_O_5_ (3 nm) nanofiller for Nb_2_O_5_-PVDF-HFP CPEs. Nb^5+^ acted as a Lewis acid center that could release more free charge carriers by interacting with the SO^2−^ group in TFSI^−^. The ionic conductivity of Nb_2_O_5_-PVDF-HFP CPEs was 6.6 × 10^–5^ S cm^−1^. Sun et al. [132] also confirmed that Al_2_O_3_ and BaTiO_3_ inorganic fillers can effectively enhance the carrier concentration in CPEs, which increased the ionic conductivity of the CPEs.

Ideally, all lithium ions are complexed with the polymers. Therefore, both anions and cations are carriers. Unfortunately, as the concentration increases, the lithium salt hardly dissociates due to the electrostatic effect. Inorganic fillers in polymers can increase the concentration of carriers in the composite system. In addition, some inorganic fillers that contain lithium sources can also contribute to carriers.

(B) Increasing the motion rate of carriers (*u)*

According to Eq. (1), as the ion transport rate increases, the ionic conductivity also increases. However, the strong polar groups in the polymer chains, such as –CN and –C–O–C, are able to form strong complexes with lithium ions. This lowers the movement ability of lithium ions. The main reason why inorganic fillers can increase the ion movement rate is that the special groups on the surface of inorganic fillers can coordinate with lithium ions to weaken the interactions between lithium ions and polymers to accelerate the movement of lithium ions [77, 115]. Moreover, some long-term continuous inorganic fillers can form interconnected conductive ion channels, which significantly increases the ion migration rate. In addition, the 3D ion-conductive framework can accelerate the ion transport rate.

**Fig. 10:**
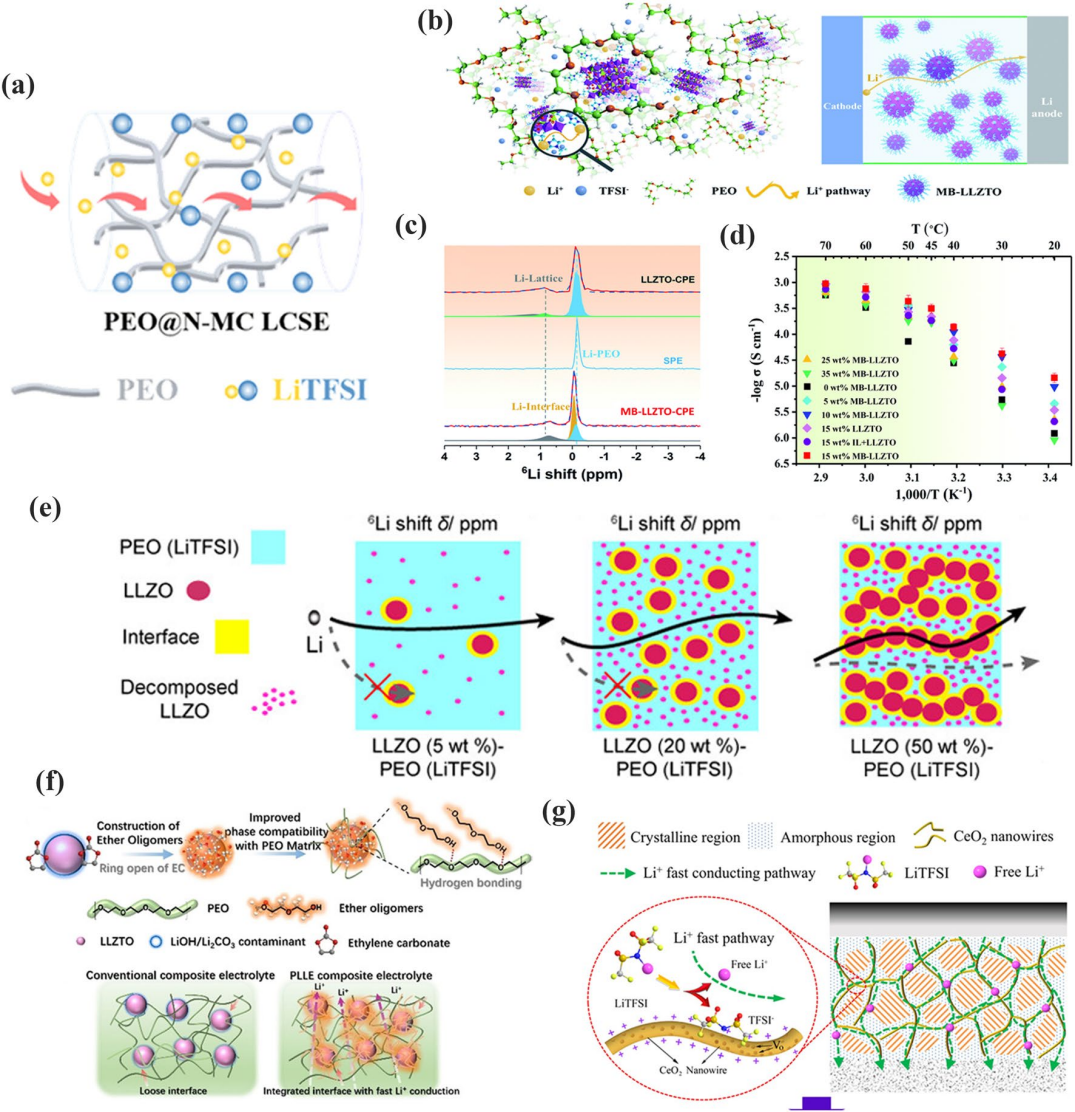
**a** Schematic diagram of the lithium-ion transfer in PEO@N-MC. Adapted with permission from Ref. [133]. **b** Figure of diffusion route of lithium ions in MB-LLZTO CPE; **c** ^6^Li NMR spectra of LLZTO CPEs and MB-LLZTO CPEs; **d** Ionic conductivity of MB-LLZTO CPEs. Adapted with permission from Ref. [100]. **e** Schematic representation of the lithium-ion route within LLZO (5–50 wt%)-PEO (LiTFSI). Adapted with permission from Ref. [135]. **f** Intermolecular interact on mechanism of LLZTO with in PEO. Adapted with permission from Ref. [136]. **g** Illustration of CeO_2_NW-CPEs. Adapted with permission from Ref. [77]

Wang et al. [133] reported an MOF functionalized with –NH_2_ for PEO@N-MC CPEs (Fig. 10a). In this case, hydrogen bonds were formed between the ether oxygen of PEO and –NH_2_, which effectively connected the adjacent MOF nanosheets. This particular interaction accelerated ion transport and promoted structural stability. The ionic conductivity of the PEO@N-MC CPEs was significantly increased by 253% compared to that of PEO-LiTFSI. Chen et al. [134] designed an inorganic filler with an MB-LLZTO molecular brush. It was introduced into PEO, as shown in Fig. 10b. The molecular brush with a special structure extends the diffusion pathway of lithium ions in MB-LLZTO PEO CPEs. As shown in Fig. 10c, on the surface of the MB-LLZTO CPEs, a third component with a value of 0.05 ppm was observed, which was introduced by the molecular brush on the LLZTO nanoparticles (Fig. 10c, bottom). Moreover, the resonance of Li in MB-LLZTO CPEs was significantly narrower than that in PEO, which suggested an irregular structure at the interface. This irregular structure provides a rapid pathway for lithium ions. Therefore, the MB-LLZTO CPEs exhibited a high ionic conductivity of 3.11 × 10^−4^ S cm^−1^ at 45 °C (Fig. 10d). Zheng et al. [135] changed the amount of inorganic filler in the polymer matrix, as presented in Fig. 10e. As LLZO content increases, the ion transfer route gradually shifts from PEO to the percolation network of interconnected LLZO particles. This continuous ion conduction channel accelerated ion transport. In Fig. 10f, Liu et al. [136] initiated the ring-opening reaction of ethylene carbonate (EC) on the LLZTO surface to form oligomers containing ether-oxygen chains. This oligomer provided an ultra-dense and fast conduction pathway for lithium ions between LLZTO and PEO substrates. The delicate design endowed LLZTO-PEO CPEs with a high ionic conductivity of 1.43 × 10^–3^ S cm^−1^. Tian et al. [77] filled CeO_2_ nanowires with SPEs, as shown in Fig. 10g. The CeO_2_ nanowires produced extended continuous ion transfer pathways, which further improved the ionic conductivity (1.1 × 10^–3^ S cm^−1^ at 60 °C).

The addition of such a surface-functionalized inorganic filler contributes to the formation of a fast lithium-ion pathway. Therefore, the inorganic filler which has a high specific surface area allows more flow area. The more continuous the ion conduction path is, the faster the ion transfer. However, some nanofillers may lead to serious phase separation, resulting in a lower ion transfer rate and a negative increase in ionic conductivity. This interfacial effect of ionic conductivity depends on the size, shape and content of the embedded filler and the relevant filler/polymer interfacial region.

#### ***Lithium-Ion Transference Number t***_***Li***_^+^

The t_Li_^+^ is another vital parameter of CPEs, which reflects the contribution of lithium ions to the total ionic conductivity. Both lithium ions and anions can move in the battery, but the anions prefer to migrate in the opposite direction to the lithium ions. Consequently, a large concentration gradient of lithium ions is formed, which blocks lithium-ion transport and produces uneven lithium-ion deposition. The relevant theoretical calculations suggest that the higher t_Li_^+^ is, the more uniform the lithium deposition. In this way, the generation of lithium dendrites can be avoided [137, 138]. However, t_Li_^+^ of CPEs is only 0.1–0.2. The calculation formula is as follows [139]:

2 $${\text{t}}_{{\text{L}}{\text{i}}^{+}}\text{=}\frac{{\text{I}}_{\text{s}}\text{(}{\text{V}}-{\text{I}}_{0}{{\text{R}}}_{0}\text{)}}{{\text{I}}_{0}\text{(}{\text{V}}-{\text{I}}_{\text{s}}{{\text{R}}}_{\text{s}}\text{)}}$$

As illustrated in Eq. (2), *R*_0_ and *I*_0_ are the initial interfacial impedance and the first current response of the cells, respectively. *R*s and *I*s are the interfacial impedance and current, respectively. *V* is the potential used for constant-potential polarization.

**Fig. 11:**
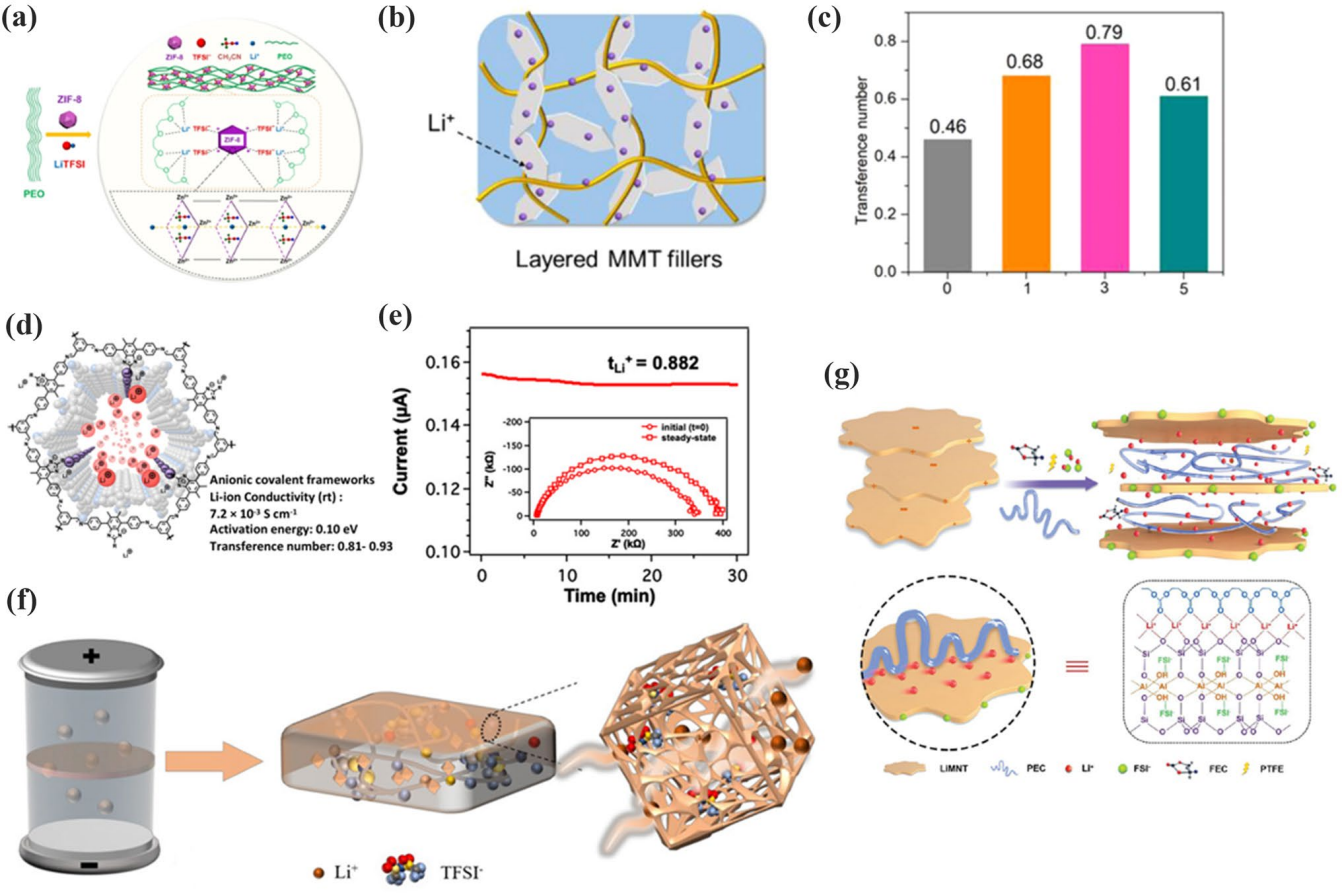
**a** Lithium-ion conductive mechanism of ZIF-8-PEO CPEs. Adapted with permission from Ref. [79].** b** Transport pathway of lithium-ion in BNN-CPEs; **c** transference number of different content of BNN. Adapted with permission from Ref. [140]. **d** Li-ion transfer in Li-ImCOFs; **e** t_Li_^+^ of LiImCOF-PEO CPEs. Adapted with permission from Ref. [141]. **f** Mechanism of ion transport in Fe-MOFs-PEO CPEs. Adapted with permission from Ref. [142]. **g** Intercalation LiMNT-PEC CPEs with enhanced t_Li_^+^ mechanism. Adapted with permission from Ref. [143]

ZIF-8-PEO CPEs were prepared by Wang et al. [79], as shown in Fig. 11a. ZIF-8, which has a surface with an abundance of Lewis acid sites, has a strong interaction with TFSI^−^. It can inhibit the movement of anion and decrease concentration polarization, resulting in a high t_Li_^+^ of 0.35. Wang and coworkers [140] reported BNN-PEGDA-MPEGA CPEs prepared with 2D boron nitride nanosheets (BNN) as inorganic nanofillers, as shown in Fig. 11b. The interpenetrating network of BNNs efficiently blocked anions. It exhibited an excellent t_Li_^+^ of 0.79, as illustrated in Fig. 11c. Zhang et al. [141] studied a series of single ion-conducted ICOFs based on imidazolium, as presented in Fig. 11d. The negatively charged groups within the ICOFs shielded the anions and permitted only lithium ions to migrate. Therefore, it showed a high t_Li_^+^ of 0.81 in Fig. 11e. Shi et al. [142] used Fe-MOFs to optimize the electrochemical properties of SPEs (Fig. 11f). The ultrafine pores of Fe-MOFs block down the anions and the free lithium-ion concentration is increased. The t_Li_^+^ increased to 0.6. Moreover, the Lewis acid–base interactions between PEO and Fe-MOFs enhanced the lithium-ion migration rate. Thus, the Fe-MOFs-PEO CPEs displayed an appreciable ionic conductivity of 2.3 × 10^–5^ S cm^−1^. Zhang et al. [143] prepared LiMNT-PEC CPEs by mixing layer-structured lithium montmorillonite (LiMNT) with PEC. 2D LiMNT has an enriched Lewis acid center that anchors the anion and releases more lithium ions, as illustrated in Fig. 11g. The intercalation structure released lithium ions rapidly, allowing the t_Li_^+^ of the LiMNT-PEC CPEs to increase to 0.83.

In general, t_Li_^+^ increases mainly due to the improvement in lithium-ion mobility, the decrease in anion mobility, or both. Inorganic fillers may increase t_Li_^+^ by immobilizing anions through abundant Lewis acid sites. In addition, the t_Li_^+^ can also be boosted by the special structures of CPEs. For example, ceramic/polymer/ceramic CPEs use a ceramic layer to block the transport of anions.

### Interactions Between Fillers and Polymers

As mentioned previously, fillers can significantly increase the ionic conductivity and t_Li_^+^ of CPEs by interacting with lithium salts. However, the transport of lithium ion is mainly dependent on polymer chain segments. However, polymers exhibit a semicrystalline aggregated structure at RT. Spherulites are the most common crystal form of polymers [144]. The behavior of ion conduction in CPEs is strongly influenced by this aggregated structure. The most evident change is ionic conductivity. Introducing inorganic fillers into polymers has proven to be an effective method for decreasing the crystalline regions of polymers and improving ionic conductivity. Therefore, the effects of fillers on the aggregated structures of polymers are mainly reflected in the changes in crystallinity Xc, glass transition temperature Tg and spherulite shape [145]. In the following chapters, we will discuss the effects of inorganic fillers on the aggregated structures of polymers in terms of these three factors.

#### Glass Transition Temperature Tg

Tg is an important parameter for the motion of polymer chain segments. Below the Tg, molecules, atoms or groups vibrate only at their respective equilibrium positions. The polymer chains are frozen, and the molecules can hardly flow. When *T* > Tg, the polymer segments begin to move but the molecular chains do not. The migration of lithium ions in polymers matrix happens mostly in the amorphous phase, while migration in the crystalline phase is limited. A majority of polymers are semicrystalline in character. Such polymers have a high Tg. It leads to a decrease in the amorphous region of the polymer, which has a detrimental effect on ion migration. Therefore, desirable electrolyte materials should exhibit at least two characteristics: a high amorphous ratio and low Tg. A number of recent studies suggested that the addition of nano additives into a polymer matrix can reduce the Tg.

Li et al. [146] designed SiO_2_-Cs-PEO CPEs for high-performance CPEs. With the increasing SiO_2_, Tg of the SiO_2_-Cs-PEO CPEs (1–4 wt% SiO_2_) decreased to − 40.5, − 41.2, − 43 and − 41.7 °C, respectively. It is evident that the introduction of SiO_2_ may increase the amorphous phase in the polymer matrix. In addition, it facilitates the movement of polymer chains, which provides a significant increase in ionic conductivity. Guo et al. [147] first introduced hydroxide (2D LDH) nanosheets into PEO. These 2D LDH fillers were rich in hydroxide radicals, forming hydrogen bonds with PEO chains to inhibit them toward the crystalline phase. Therefore, the 2D LDH-PEO CPEs showed a decrease in Tg. Xie et al. [81] doped ZnO quantum dots into PEO by vapor phase infiltration (VPI). The Tg of ZnO-PEO CPEs was significantly reduced to − 37.6 °C (compared to − 34.8 °C for PEO-LiTFSI). Guo et al. [148] prepared ZIF-67-PEO CPEs. Compared to PEO-LiTFSI (− 37.6 °C), the ZIF-67-PEO CPEs showed a significant decrease in Tg (− 40.0 °C).

Inorganic fillers are advantageous for reducing the Tg of CPEs mainly for the following reasons:

1. The polar groups in the polymer molecule may lead to the high rigidity of the molecular chain segments. However, the interaction between inorganic fillers and polymer chain segments can lower the intermolecular forces and enhance the motion of the polymer chains.
2. Inorganic filler, as a small molecule plasticizer, can increase the flexibility of polymer molecular chains.

#### Degree of Crystallinity Xc

It is widely believed that ionic conduction happens mostly in the amorphous. The crystallization process of polymers involves two processes: nucleation and crystal growth. Nuclei are formed in the nanoregions of polymer chain segments and then further separated or grown. Xc is the degree of long-range ordering of the polymer chains. In CPEs, inorganic fillers act as a solid plasticizer to disrupt the orderly arrangement of polymer chains, thereby reducing the crystallinity of the polymer.

**Fig. 12:**
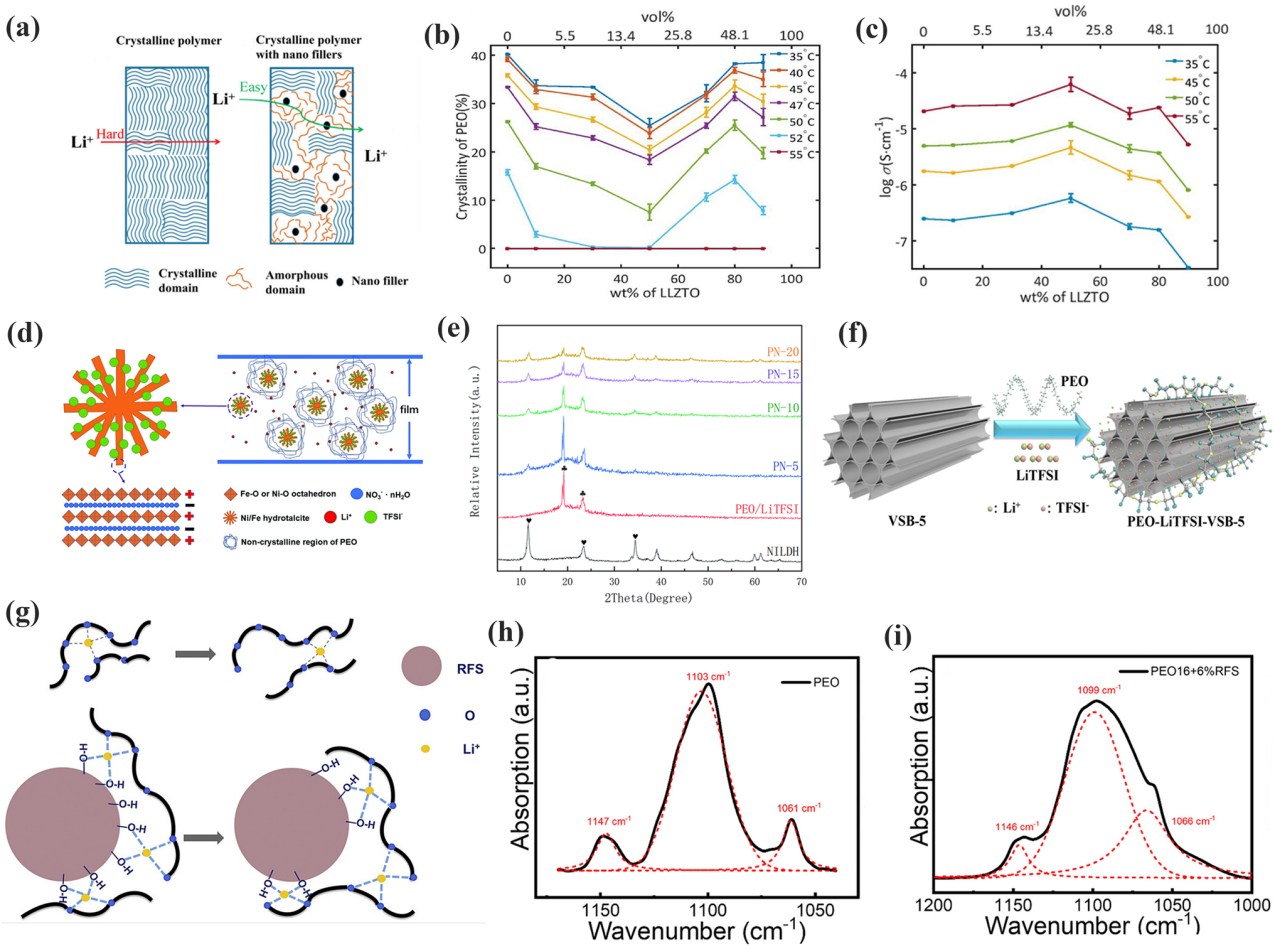
**a** Schematic illustration of the lithium-ion transfer across crystalline polymer and crystalline polymer with nanofillers; **b** Xc of PEO on LLZTO contents; **c** the dependence of ionic conductivity on LLZTO contents. Adapted with permission from Ref. [149]. **d** Structure sketch of NILDH-PEO CPEs improving the ionic conductivity; **e** XRD patterns of NILDH and NILDH-PEO CPEs. Adapted with permission from Ref. [150]. **f** VSB-5-enhanced SPEs for lithium battery. Adapted with permission from Ref. [151]. **g** Schematic diagram of RFS effect the lithium-ion conduction; **h-i** Attenuated total reflection infrared spectra of PEO and PEO16-RFS. Adapted with permission from Ref. [152]

As shown in Fig. 12a, fillers decrease Xc by disrupting the ordered structure of the polymer. A systematic study of the relation between the crystallinity and ionic conductivity of PEO was conducted by Bo et al. [149]. As shown in Fig. 12b, the Xc of PEO first decreased with increasing LLZTO. The crystallinity of LLZTO-PEO CPEs reached the minimum value when the addition of LLZTO was 50 wt%. Unexpectedly, after continuing to increase LLZTO, the Xc was increased. The consequence may be associated with the spatial distribution of LLZTO particles in the PEO substrates. Moreover, with the increase in LLZTO, a maximum ionic conductivity of LLZTO- PEO CPEs was obtained with 50 wt% LLZTO, then it started to decrease, as shown in Fig. 12c. The ionic conductivity of LLZTO-PEO CPEs showed a completely opposite trend to that of the crystallinity. This work suggests a possible relationship between the Xc and the ionic conductivity of CPEs. Yang et al. [150] introduced nickel–iron-based layered hydroxide (NILDH) into the polymer matrix to reduce the crystallinity of NILDH-PEO CPEs (Fig. 12d). It can be observed that the intensity of the characteristic diffraction peak of PEO gradually decreases with the increase in NILDH (Fig. 12e). The NILDH particles disrupt the normal organization of PEO chains and prevent the crystallization. As illustrated in Fig. 12f, Guan et al. [151] used the hydrogen bonding effect between nickel phosphate (VSB-5) nanorods and PEO to disorder polymer chains in VSB-5-PEO CPEs. Wang et al. [152] doped phenolic resin nanospheres (RFS) fillers into PEO-LiClO_4_ to investigate the effect of RFS on Xc, as shown in Fig. 12g. The surface groups (-OH) of RFS interacted with the PEO through hydrogen bonding. As shown in Fig. 12h, PEO showed distinct C–O–C stretching vibrations at 1103, 1147 and 1061 cm^−1^. When the RFS filler was doped, the C–O–C in the amorphous region was shifted from 1096 to 1099 cm^−1^, as illustrated in Fig. 12i. That is, the addition of filler changed the conformation of PEO to increase the amorphous region.

Semicrystalline polymers usually present a low ionic conductivity (10^–8^–10^–6^ S cm^−1^) due to the high Xc of the polymer matrix. Therefore, in addition to inorganic fillers reducing Xc, there are two common methods:

1. Modifying the polymer by grafting to reduce the degree of regularity of the molecular chains.
2. Adding organic plasticizers into the polymer decreases the intermolecular interactions and increase the flexibility of the molecular chains.

Although the above two approaches can effectively reduce the crystallinity of CPEs, it would sacrifice the mechanical strength. Accordingly, it is necessary to achieve a compromise between Xc and mechanical strength in the following work.

#### Effective of Spherulites for Polymers

**Fig. 13:**
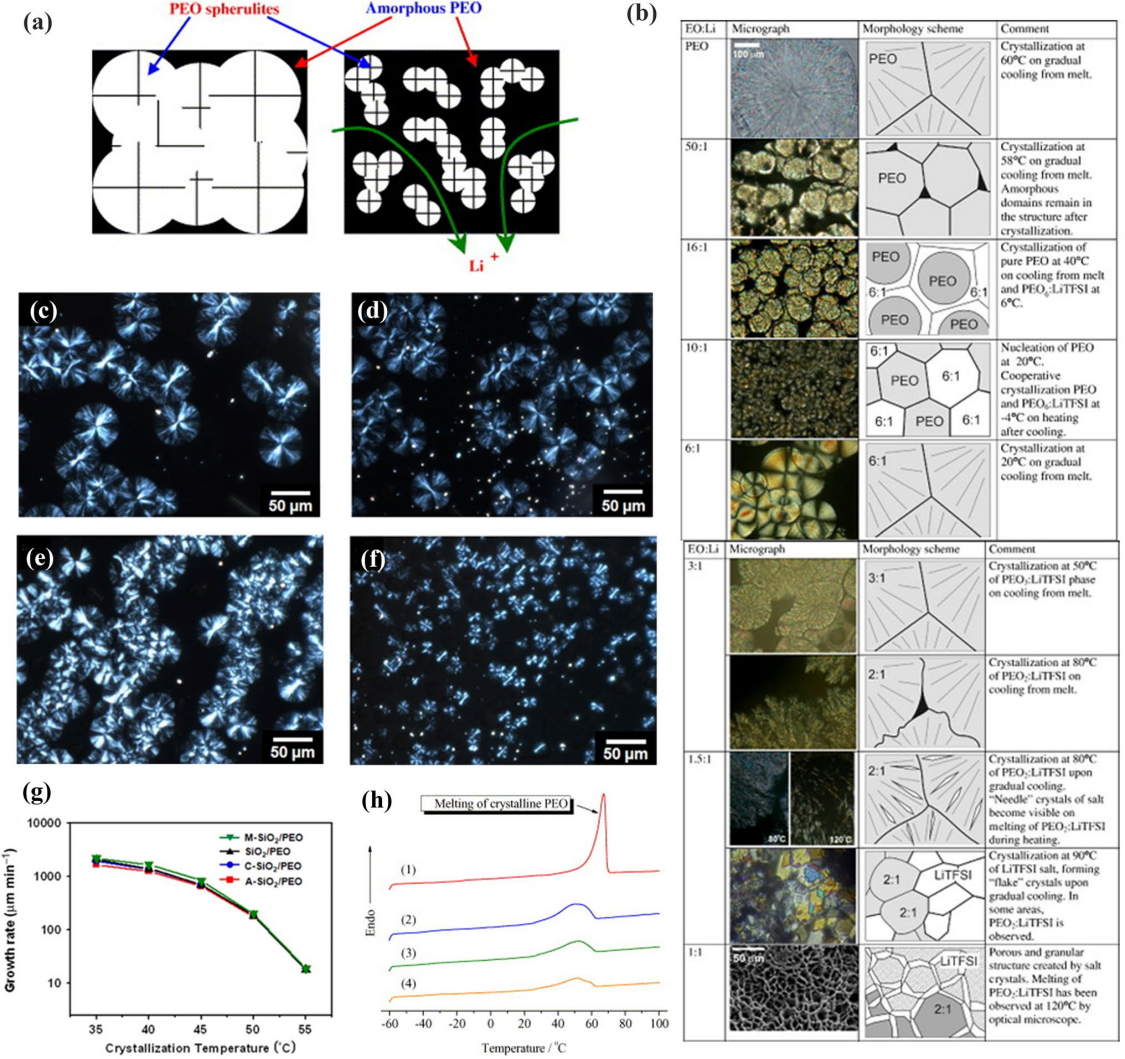
**a** Illustration of the transport of lithium ions in PEO spherites. Adapted with permission from Ref. [153]. **b** The results for PEO/LiTFSI electrolytes of different content salt. Adapted with permission from Ref. [154]. **c–f** POM pictures of PEO with neat SiO_2_, M–SiO_2_, C-SiO_2_ and A-SiO_2_; **g** Log plot of the spherites growth rate of SiO2-PEO composites versus the crystallization temperature of as a function of SiO_2_ content. Adapted with permission from Ref. [156]. **h** DSC plots of PEO10-LiClO_4_/10%ZSM-5. Adapted with permission from Ref. [157]

Crystalline polymers mainly show many spherulites. Spherulite is spherical in shape and varies in size from micrometers to a few millimeters. Figure 13a [153] shows a transport schematic of lithium ions in PEO. Large spherulites stacked with one another that makes the diffusion of lithium ions difficult. When the spherulites become small, the amorphous area increases, and the diffusion of lithium ions is accelerated. Marzantowicz [154] reported that the morphology of spherulites varied with different EO/Li ratios (Fig. 13b). When the concentration of lithium salt was low (EO/Li = 50), the crystalline phase of PEO mainly dominated. When EO/Li = 6, the spherulites became small. The crystalline region was clearly distinguished from the amorphous region. However, high concentrations of salt led to severe phase separation.

Choi et al. [155] found that different sizes of Fe_3_O_4_ nanoparticles had completely different effects on the aggregated state of PEO. The small size of Fe_3_O_4_ (0.023 µm, 10 wt%) produced more nucleation sites, which led to smaller spherulites, resulting in a decrease in crystallinity. However, Fe_3_O_4_ (5 µm, 10 wt%) produced significantly larger spherulites due to fewer nucleation sites, and the Xc was higher than that of Fe_3_O_4_ (0.023 µm, 10 wt%). Jang et al. [156] analyzed the effect of different surface modifications of SiO_2_ nanofillers on spherulites, including SiO_2_ (Fig. 13c), methoxy-treated SiO_2_ (M–SiO_2_, Fig. 13d), carboxylate-treated SiO_2_ (C-SiO_2_, Fig. 13e) and amine-treated SiO_2_ (A-SiO_2_, Fig. 13f). The high nucleation densities of C-SiO_2_ and A-SiO_2_ led to a smaller spherulite size. This may be attributed to the electrostatic force between the strong polar groups (on the SiO_2_ surface) and the PEO segments. This interaction affects the migration of the polymer chains to the crystalline surface, which results in a lower crystallinity. Furthermore, the interaction between the M-SiO_2_ and PEO segments was relatively weak, thus resulting in a higher Xc growth rate (Fig. 13g). Qiu et al. [157] compared the influence of Al_2_O_3_ and ZSM-5 on Xc. The number of PEO spherulites further increased with the incorporation of Al_2_O_3_ and ZSM-5. And the radius of spherulites decreased to about 20 μ m on average. The melt enthalpy (∆Hm) and Xc were both affected, as shown in Fig. 13h. The ionic conductivity increased from 1.5 × 10^–7^ S cm^−1^ (PEO10-LiClO_4_) to 1.4 × 10^–5^ S cm^−1^ (PEO10-LiClO_4_/10%ZSM-5).

The ionic conductivity of CPEs is complicated by the presence of both crystalline and noncrystalline phases below the Tm. The morphology and number of spherulites are related to Xc. In general, the large number of nucleation sites formed by inorganic fillers in polymers increases the number of spherulites significantly. However, the size of spherulites decreases rapidly. In this procedure, the amorphous region increases, which accelerates the conduction of lithium ions.

From the above analysis, it is clear that the interactions among the inorganic filler, lithium salt and polymer matrix have important effects on the electrochemical properties of CPEs. At present, it is widely assumed that the addition of inorganic fillers can enhance the electrochemical properties of CPEs, which is mainly reflected by an increased ionic conductivity and t_Li_^+^. However, this process involves several factors, including carrier concentration, ion migration rate, Tg and Xc. Despite inorganic fillers enhancing the electrochemical and mechanical characteristics of CPEs, aggregation in the polymer matrix and compatibility with the electrode are still major obstacles to practical applications.

## Interface Between CPEs and Electrodes

Although the migration of lithium ions in the bulk of CPEs has been addressed, lithium-ion conduction should not be neglected at the electrode–electrolyte interface. The ion conduction at the electrode interface is quite different from that in the bulk phase of CPEs. In addition, the stability of the interface between electrolyte and electrode remains a bottleneck of SSLBs. The interfacial stability is determined by poor electrolyte–electrode contact, lithium dendrite growth and high-pressure decomposition [27]. To solve these problems, CPEs with the advantages of two components (organic and inorganic) become popular in recent years [158, 159].

**Fig. 14:**
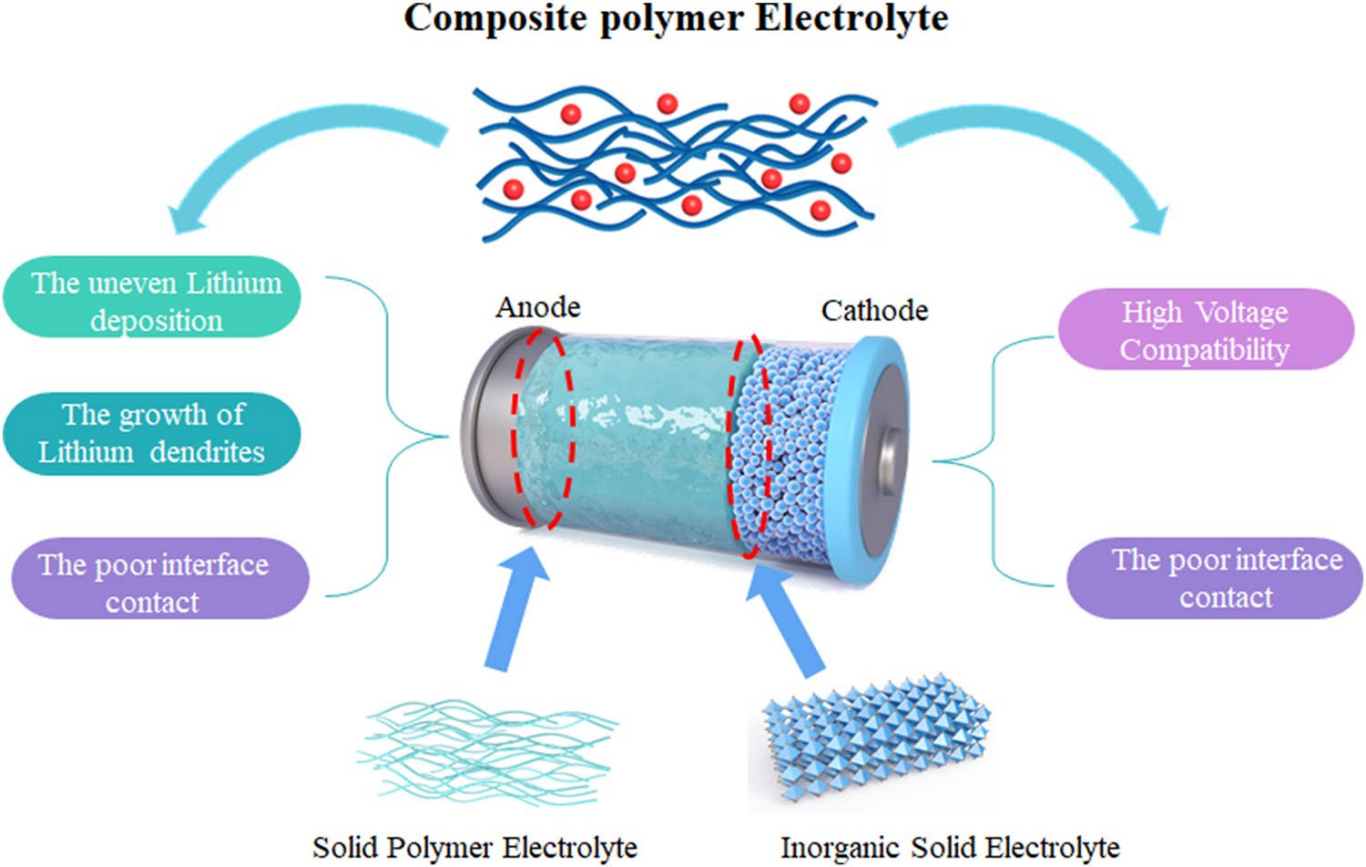
Relationship between composite polymer electrolytes and anode and cathode

In this section, we will discuss the improvement in the interfacial stability between CPEs and electrodes in terms of CPEs stabilizing the cathode and CPEs stabilizing the anode. The relationship between CPEs and anode and cathode is depicted in Fig. 14.

### Modifications of the CPE/Cathode Interface

**Fig. 15:**
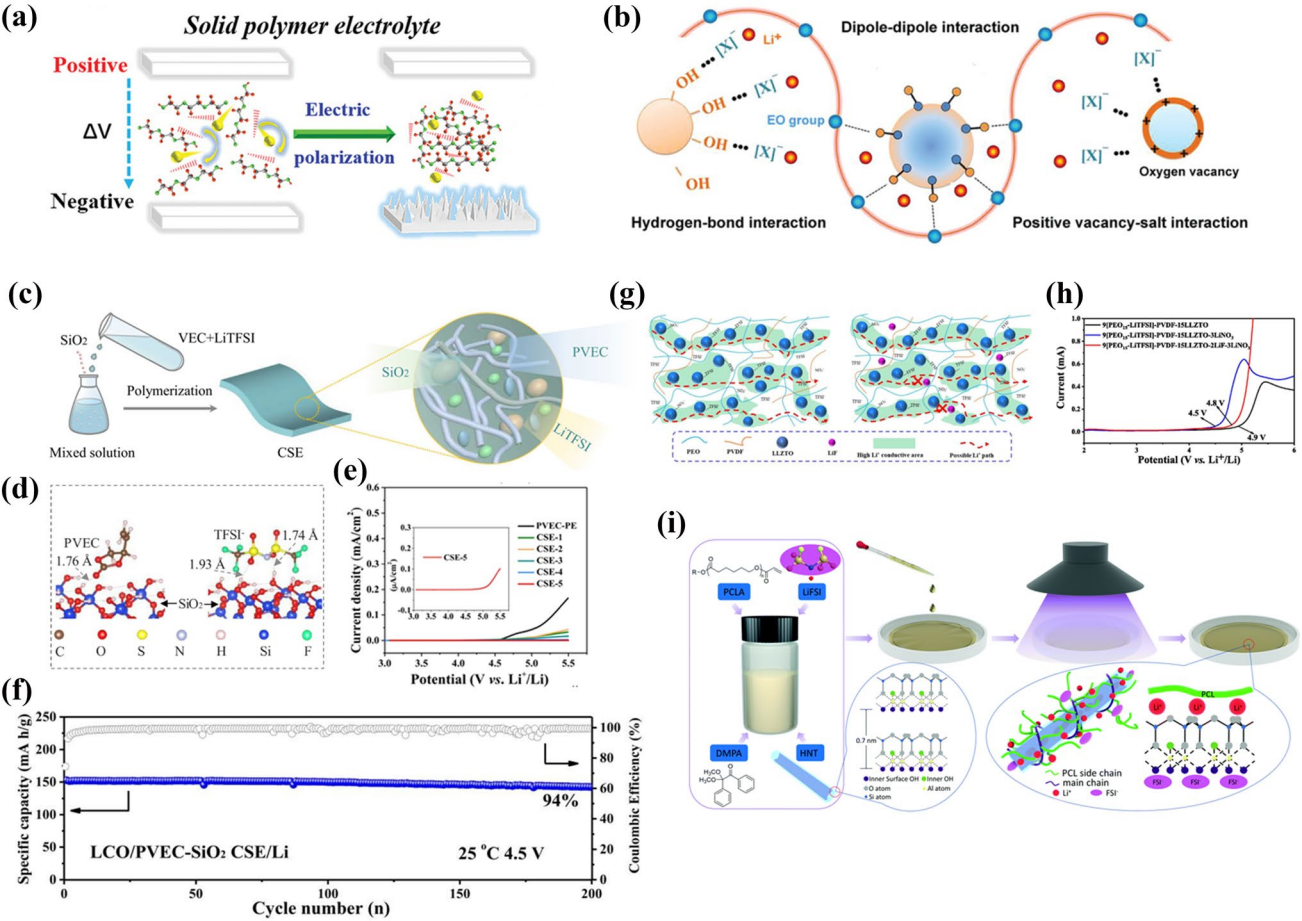
**a** Schematic for the electrochemical attenuation with the electric field. Adapted with permission from Ref. [160]. **b** Lewis acid–base interactions between inorganic additives and polymers. Adapted with permission from Ref. [36]. **c** Schematic diagram of preparing the PVEC-SiO_2_ CPEs; **d** Intermolecular interaction in PVEC-SiO_2_ CPEs by DFT; **e** electrochemical stability window of PVEC-SiO_2_ CPEs; **f** cycling stability of PVEC-SiO_2_ CPEs. Adapted with permission from Ref. [161]. **g** Diagram of lithium-ion conductive pathways without and with LiF additive; **h** Comparison of LSV results of LLZTO-PEO/PVDF CSEs with different additives. Adapted with permission from Ref. [162]. **i** Schematic illustration of the preparation of the HNTs-PCL CPEs. Adapted with permission from Ref. [163]

Under an electric field, electrolytes generate many polarization domains due to intermolecular forces, resulting in the deterioration of electrochemical properties (Fig. 15a) [160]. Thermodynamically, high-voltage compatibility of the electrolyte indicates the ability to resist oxidative decomposition. The highest occupied molecular orbital (HOMO) of all components in the electrolyte (polymers, lithium salts, additives, etc.) must be lower than that of the cathode. Inorganic fillers improve the electrochemical stability of CPEs through Lewis acid–base interactions (hydrogen bonding, vacancy and dipole–dipole interactions) with polymers and lithium salts (Fig. 15b) [36].

Wang et al. [161] prepared CPEs with a wide electrochemical stability window and high ionic conductivity by admixing SiO_2_ nanoparticles into polyvinyl ethylene carbonate (PVEC) (Fig. 15c). Theoretical calculations and experimental results confirmed that the enhancement of the antioxidant capacity of SiO_2_-PVEC CPEs was mainly attributed to hydrogen bonds. As shown in Fig. 15d, the H atoms on the surface of SiO_2_ and the O atoms (C=O and O=S=O) in PVEC and TFSI^−^ formed hydrogen bonds. The local intermolecular interaction increased the antioxidant capacity of the SiO_2_-PVEC CPEs. As a result, the electrochemical window was up to 5.0 V, as in Fig. 15e. LCO|SiO_2_-PVEC CPEs|Li cells provide favorable cycle stability with about 94% capacity retention at a cutoff voltage of 4.5 V (Fig. 15f). In the work of Li et al. [162], LiF as a synergistic additive was added to LLZTO-PEO CPEs to improve the electrochemical stability at a high cutoff voltage (Fig. 15g). Due to the dipole–dipole interactions between LiF and PEO, the electron-hopping energy level of PEO changed to increase the oxidative decomposition potential of PEO. As depicted in Fig. 15h, the oxidative decomposition potential of the LLZTO-PEO/PVDF CPEs increased to 4.8 V. Xu et al. [163] prepared high-voltage compatible CPEs consisting of HNTs and PCL by an in situ technique (Fig. 15i). The external surface of the HNTs was negatively charged, while the internal surface was positively charged. The Lewis acid–base interactions between the HNTs and polymers induced changes in the electron-hopping energy levels of the polymer, thereby enhancing the high voltage resistance of the HNTs-PCL CPEs. These HNTs-PCL CPEs exhibited a potential window of 5.1 V.

In addition, a number of inorganic fillers with oxygen vacancies were effective in enhancing the high-voltage stability of CPEs. Kang et al. [164] introduced Gd–CeO_2_ nanowire into PEO to prepare Gd–CeO_2_-PEO CPEs. Benefiting from the abundant oxygen vacancies on the surface of Gd–CeO_2_, the electrochemical window of Gd–CeO_2_-PEO CPEs was increased to 5.0 V (vs. PEO-LITFSI at 4 V), and the ionic conductivity was increased 5 × 10^–4^ S cm^−1^. NCATP (Ce-NASICO) was synthesized by Huang et al. [165]. NCATP enabled the electrolyte to exhibit an excellent antioxidant capacity (5 V) by influencing the electron-hopping energy level of PVDF-HFP. MoO_3_-PEO CPEs were prepared by Wang et al. [166]. The abundant lattice oxygen on the surface of MoO_3_ showed a certain adsorption effect on the PEO segments, which stabilized the PEO chain structure and inhibited the decomposition of PEO chains under high voltage.

Inorganic fillers can improve the antioxidant capacity of CPEs. This is mainly reflected in the effect on the electron-hopping energy levels of the polymer. On the one hand, inorganic fillers are enriched with polar groups (–OH, –COOH, etc.) by grafting which can stabilize the polymer matrix. On the other hand, the elemental doping of inorganic fillers increases surface defects. These defects can stabilize the lithium salt from which the electrochemical stability of the electrolyte is enhanced.

### Modifications of the CPE/Anode Interface

As the “holy grail” of high-performance solid-state cells, lithium metal is one of the most promising anodes. However, interface problems between lithium metal and CPEs still remain. The problems of lithium metal are mainly related to two aspects:

1. During the periodic cycling of the battery, the expansion and shrinkage of the lithium metal lead to a poor contact.
2. Unstable ion transport behavior leads to uneven lithium deposition and thus to the formation of lithium dendrites [167, 168].

The growth of lithium dendrites may puncture the electrolyte, resulting in contact between cathodes and anodes. Recent work has demonstrated that the compatibility of the solid-state electrolyte with the anode can also be improved effectively by the incorporation of inorganic fillers. The roles played by inorganic fillers in alleviating the interface problems are as follows: first, the inorganic filler can homogenize the lithium flux by regulating the ion transport behavior in the electrolyte bulk phase. Thus, the lithium dendrite generation can be controlled at the origin. Second, the inorganic filler can significantly reinforce the mechanical strength of CPEs to suppress the growth of lithium dendrites.

#### Regulation of Lithium-Ion Deposition

Thermodynamically, lithium dendrites originate from the nucleation of lithium dendrites due to uneven local current densities. Therefore, the structural design of CPEs is beneficial for reducing the effective current density. In particular, some 3D inorganic fillers can accelerate ion transport and reduce the space charge density to slow down the formation of lithium dendrites [11].

**Fig. 16:**
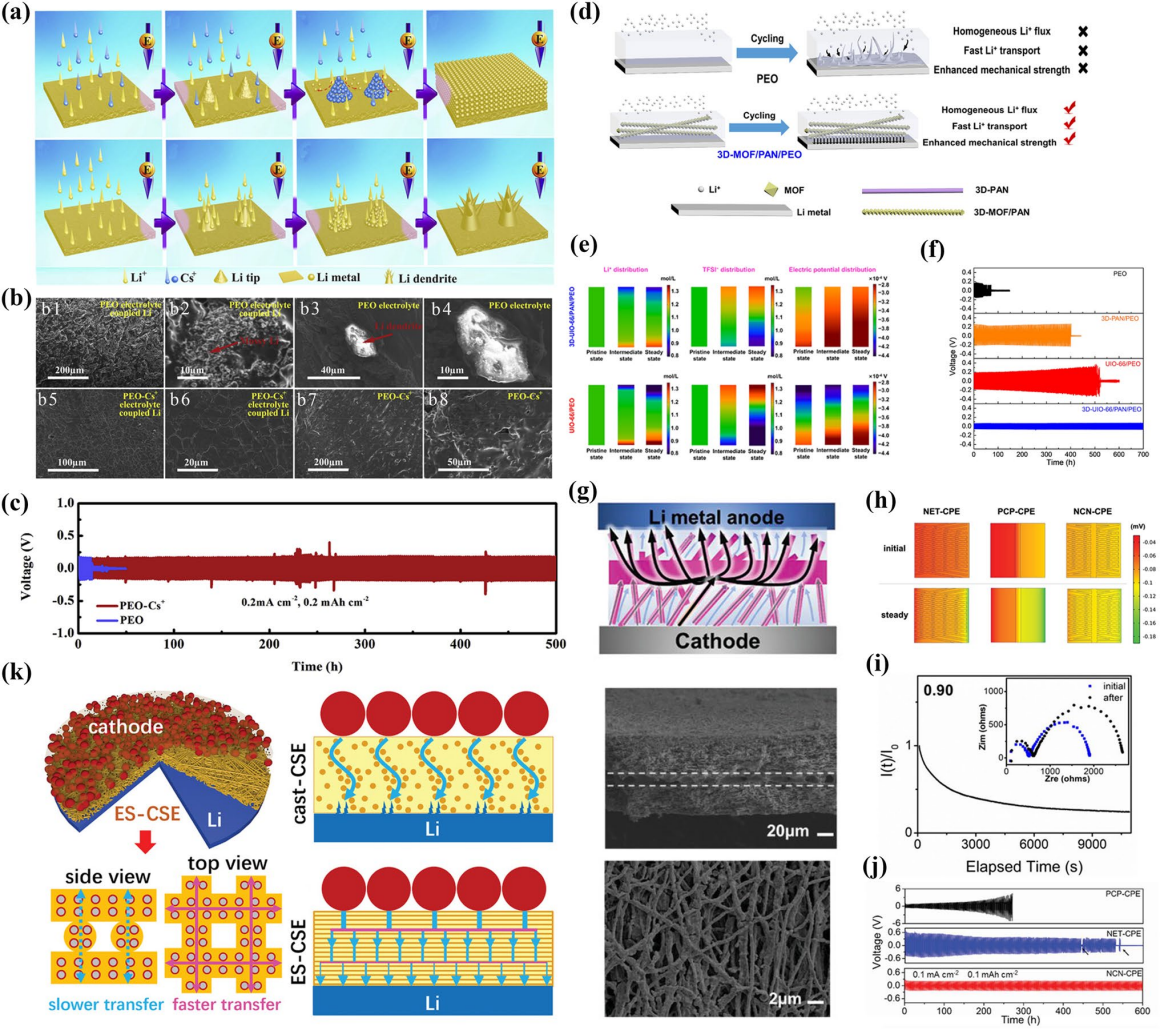
**a** Illustration of the Li deposition process for PEO-Cs^+^ and conventional PEO electrolyte;** b** SEM images of (b1–b4) PEO SPEs after 100 h; SEM images of (b5–b8) CsClO_4_-PEO CPEs after 100 h; **c** cycling stability of the Li||Li symmetrical cells assembled with CsClO_4_-PEO CPEs and PEO SPEs. Adapted with permission from Ref. [170].** d** Schematic diagram of the growth of Li dendrites in PEO and 3D-MOF/PAN/PEO; **e** the COMSOL simulation for Li^+^, TFSI^−^ and potential distribution of UIO-66/PEO and 3D-UIO-66/PAN/PEO; **f** long-term cycle reliability of symmetric Li|3D-UIO-66/PAN/PEO CPEs|Li cells. Adapted with permission from Ref. [171]. **g** Schematic diagram of lithium-ion transport NCN-PEO CPEs and the side view and top view of NCN-PEO CPEs; **h** FEM simulations of electric potential distribution in NET-PEO CPEs, PCP-PEO CPEs and NCN-PEO CPE; **i** lithium-ion transference number of NCN-PEO CPEs; **j** Li plating/stripping test with a constant current density of 0.1 mA cm.^−2^. Adapted with permission from Ref. [172]. **k** Schematic illustration of LLZO-PEO CPEs works in solid-state Li metal batteries. Adapted with permission from Ref. [173]

An anion-immobilized LLZTO-PEO CPE was proposed by Zhang et al. [169]. Compared with conventional liquid electrolytes, LLZTO-PEO CPEs can bundle anions to induce a uniform distribution of lithium ions. Sun et al. [170] proposed a self-healing electrostatic shielding strategy to achieve uniform lithium-ion deposition in PEO-based electrolytes. As shown in Fig. 16a, homogeneous lithium-ion deposition was accomplished by introducing CsClO_4_ (0.05 M). Interestingly, Cs^+^ showed a lower reduction potential than lithium ions (1.7 mol L^−1^). Different from the conventional CPEs, Cs^+^ initially formed a positively charged electrostatic shield coating around the lithium tip during lithium deposition. This forced the lithium ions to be deposited preferentially in the neighboring region of Cs^+^. Finally, a smooth deposition layer and a dendrite-free lithium anode surface were obtained. After 100 h of cycling, a large amount of mossy lithium was observed on the anode when coupled with PEO SPEs (Fig. 16b1, b2). In addition, some large lithium dendrites in 10–20 μm were also observed on the surface of PEO SPEs in (Fig. 16b3, b4). However, for CsClO_4_-PEO CPEs, no lithium dendrites or mossy lithium were observed on the anode (Fig. 16b5, b6). Moreover, the original morphology of CsClO_4_-PEO CPEs was maintained. Thus, the CsClO_4_-PEO CPEs benefitted from the low potential to achieve uniform lithium deposition. The Li|CsClO_4_-PEO CPEs|Li battery realized stable plating/exfoliation performance for 500 h at 0.2 mA cm^−2^ (Fig. 16c). Cai et al. [171] exploited a network of interconnected 3D-UIO-66-PAN/PEO CPEs to homogenize lithium-ion fluxes. As shown in Fig. 16d, the uniform distribution of UIO-66 on nanofibers favored the creation of a continuous ion transport pathway, which facilitated lithium-ion transport. Moreover, UIO-66 with a moderate pore size and strong cationic sites allowed a uniform lithium flux distribution by limiting anion transport. In Fig. 16e, the COMSOL result reveals that 3D-UIO-66-PAN/PEO CPEs with a small concentration gradient for lithium ions and TFSI^−^ ions during lithium deposition, which suggests a homogeneous lithium-ion flux. Moreover, the potential field was smaller than that of the UIO-66-PAN/PEO CPEs. Notably, due to the uniform lithium-ion flux and the fast lithium-ion transport of 3D-UIO-66-PAN/PPEO, the Li|3D-UIO-66/PAN/PEO CPEs|Li cells did not suffer from short-circuiting even after 700 h of cycling (Fig. 16f). Fan et al. [172] designed NCN-CPEs composed of corrugated 3D nanowire bulk-ceramic-nanowires (NCN) (Fig. 16g). This special NCN backbone alleviated the polarization concentration at the electrode/electrolyte interface and provided a uniform interfacial lithium-ion flux to the anode. In Fig. 16h, finite element simulation results show that the electrolyte composed of LLZTO ceramic sheets (NET-PEO CPEs) and LLZTO nanowires (PCP-PEO CPEs) suffered from a high diffusion potential barrier for lithium-ion transport due to the higher local space charge. This resulted in a nonuniform lithium-ion flux at the electrode. However, the special sandwich structure of the NCN-PEO CPEs provided a definite advantage. Notably, the NCN-PEO CPEs exhibited an excellent t_Li_^+^ of 0.9 (Fig. 16i). The Li|NCN-PEO CPEs|Li cell showed a flat voltage profile with no short-circuiting (0.1 mA cm^−2^) for 600 h (Fig. 16j). LLZO-PEO CPEs with vertical/horizontal anisotropy were prepared by Guo et al. [173]. As shown in Fig. 16k, the LLZO ultrafine fibers rapidly transferred lithium ions and reduced the uneven distribution of the electric field, thus achieving excellent electrochemical performance. Wu et al. [174] adjusted the interfacial potential distribution between the electrolyte and the anode in situ generating Li_3_P on the surface of SPEs, allowing the homogenous plating and stripping of lithium ions.

The incorporation of electronegative (vs. Li^+^) elements in the electrolyte to prevent the formation of lithium cores and the addition of porous inorganic fillers to realize a uniform lithium-ion flux are effective strategies for promoting homogeneous lithium-ion deposition and inhibiting the formation of lithium dendrites. In addition, strategies for modifying lithium metal can also achieve the same purposes.

#### Inhibition of Lithium Dendrites

ISEs have a superior shear modulus, which can strongly restrain the growth of lithium dendrites. However, ISEs suffer from high interfacial resistance. Therefore, it is difficult to balance interfacial compatibility and ionic conductivity. SPEs have excellent interface contact. But lithium dendrites can still penetrate the electrolyte and cause short-circuiting inside the cells. Therefore, enhancing the mechanical strength of SPEs is another important strategy to restrain lithium dendrites.

**Fig. 17:**
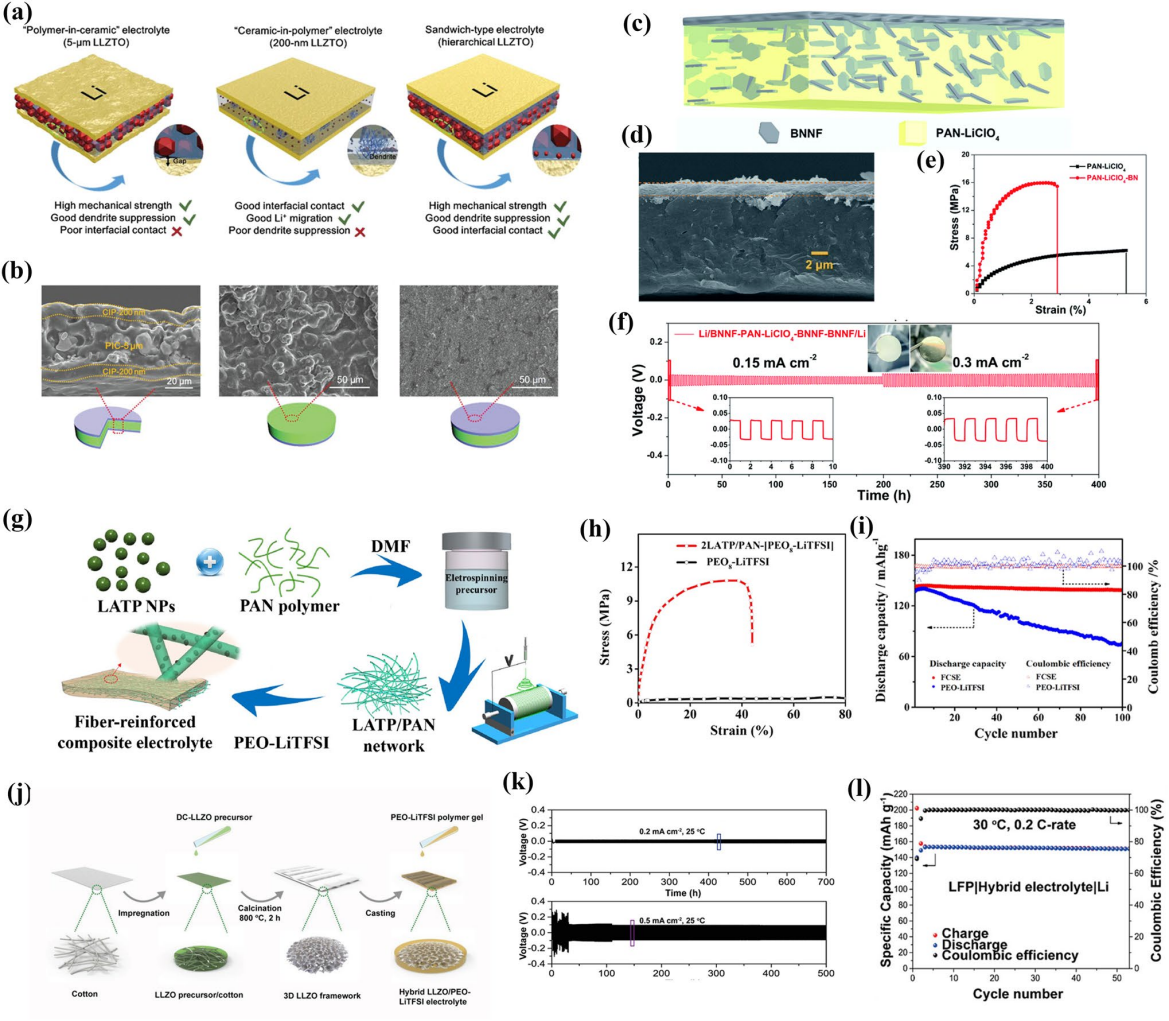
**a** Description of the PIC-5 µm, CIP-200 nm, and hierarchical CPEs; **b** cross-sectional SEM pictures of LLZTO-PEO CPEs with hierarchical structure. Adapted with permission from Ref. [175]. **c** Schematic of the BNNF-PAN-LiClO_4_-BNNF; **d** cross-sectional SEM image of the BNNF-PAN-LiClO_4_-BNNF; **e** stress–strain curves of the PAN-LiClO_4_ and BNNF-PAN-LiClO_4_-BNNF CPEs;** f** BNNF-PAN-LiClO_4_-BNNF CPEs and lithium metal anodes after cycles. Adapted with permission from Ref. [176]. **g** Schematic Illustration for the preparation of the LATP-PAN-PEO CPEs; **h** stress–strain curves of LATP-PAN-PEO CPEs and PEO8–LiTFSI; **i** cycling stability of Li|LATP-PAN-PEO CPEs|LiFePO_4_ batteries at 0.2C. Adapted with permission from Ref. [177].** j** Schematic illustration for the preparation of the LLZO-PEO CPEs. **k** Cycles of Li|LLZO-PEO CPEs|Li 0.1 and 0.2 mA cm^−^.^2^; **l** Cycling performance of LiFePO_4_|LLZO-PEO CPEs|Li at 0.2C. Adapted with permission from Ref. [178]

To balance the mechanical and electrochemical properties of CPEs, LLZTO-PEO CPEs with a sandwich structure were designed by Huo et al. [175]. Figure 17a shows these sandwich-structured LLZTO-PEO CPEs. The external layer consisted of 20%-LLZTO (200 nm) and PEO, which resulted in good interfacial contact. The intermediate layer consisted of 80%-LLZTO (5 µm) and PEO, which effectively inhibited lithium dendrites. Figure 17b shows the SEM images of the LLZTO-PEO CPEs with a hierarchical structure. With this rigid-flexible design, the Li|LLZTO-PEO CPEs|Li cell was stably maintained for 400 h at 0.2 mA cm^−2^. Jiang et al. [176] reported BNNF-PAN-BNNF CPEs, as presented in Fig. 17c. The BNNF-PAN-BNNF CPEs with a bilayer structure are shown in Fig. 17d. For these CPEs, the BNNFs endowed it with an excellent tensile strength (16.0 MPa) and Young's modulus (563.7 MPa), as shown in Fig. 17e. Due to the above advantages, the Li|BNNF-PAN- BNNF CPEs|Li cell had a small overpotential, while the lithium metal hardly changed after 400 h of cycling (Fig. 17f). Fan et al. [177] adopted a new strategy for inhibiting lithium dendrites, as illustrated in Fig. 17g. The excellent mechanical strength of the flexible network of LATP-PAN (tensile strength of 10.72 MPa in Fig. 17h) enhanced stress tolerance. Thus, LATP-PAN/PEO CPEs suppressed the development of lithium dendrites through the fiber network. Moreover, the Coulombic efficiency of the Li|LATP-PAN/PEO CPEs|LiFePO_4_ battery was maintained at 99% after 100 cycles, which indicated the excellent interfacial stability between the electrolyte and anode during the cycles (Fig. 17i). Hu et al. [178] prepared LLZO-PEO CPEs by filling a 3D conductive lithium framework (LLZO) with PEO in Fig. 17j. The LLZO-PEO CPEs not only provided a high ionic conductivity (8 × 10^–4^ S cm^−1^), but the rigid backbone structure hindered the growth of dendrites. As indicated in Fig. 17k, the Li|LLZO-PEO CPEs|Li cell did not short circuit even after 500 h cycles at 0.2 and 0.5 mA cm^−2^. Moreover, the LiFePO_4_|LLZO-PEO CPEs|Li cell maintained nearly 100% capacity in 50 cycles, as shown in Fig. 17l.

## Conclusion

The solid-state electrolyte plays a significant role in SSLBs. Currently, CPEs are regarded as a prospective solid-state electrolyte because they inherit the advantages of ISEs and SPEs. However, CPEs still need to overcome some drawbacks, for example, a low ionic conductivity and undesirable interfaces. Therefore, this review explores the contribution of inorganic fillers in improving the electrochemical performance as well as the interfacial compatibility of CPEs.

According to the transport method of lithium ions in CPEs, it is known that the important role of inorganic fillers is to increase the amorphous region of the polymer matrix and, in this way, to increase the number of movable polymer chain segments. At the macroscopic level, the changes in the polymer aggregated state structure are an important reason for the changes in the amorphous regions. Spherites are the main crystalline structure of polymers. spherites, including size and quantities. Once the aggregated structure of CPEs is changed, Xc as well as Tg will also be changed.

And at the microscopic level, the inorganic filler will induce a change in the ion transport behavior. This is due to the fact that a new interfacial phase, the polymer–filler interface, is created in CPEs. New ion transport channels will be formed at this interface. This interfacial effect can be attributed to the Lewis acid–base interaction among lithium salt–filler–polymer. The intensity of Lewis acid–base interactions is related to the species, morphology, concentration and surface properties of the inorganic filler. Besides, the special structures of inorganic fillers, such as nanowires, 3D network structures, and vertically aligned structures, can increase the u. Next, functionalized inorganic fillers, such as Lewis acid or base sites on the surface, can accelerate the dissociation of lithium salts and promote the coordination of the polymer with lithium ions. Also, Lewis acid–base interactions can increase the n in the CPEs systems. They both contribute to the ionic conductivity of the CPEs. In addition, the special characteristics of the fillers, such as the surface with positive charges, -HSO_3_, -NH_2_, -COOH, generally interact with the lithium salt in two ways: increasing the mobility of the lithium ion or limiting the motility of the anion. Those interactions all contribute to t_Li_^+^.

**Fig. 18:**
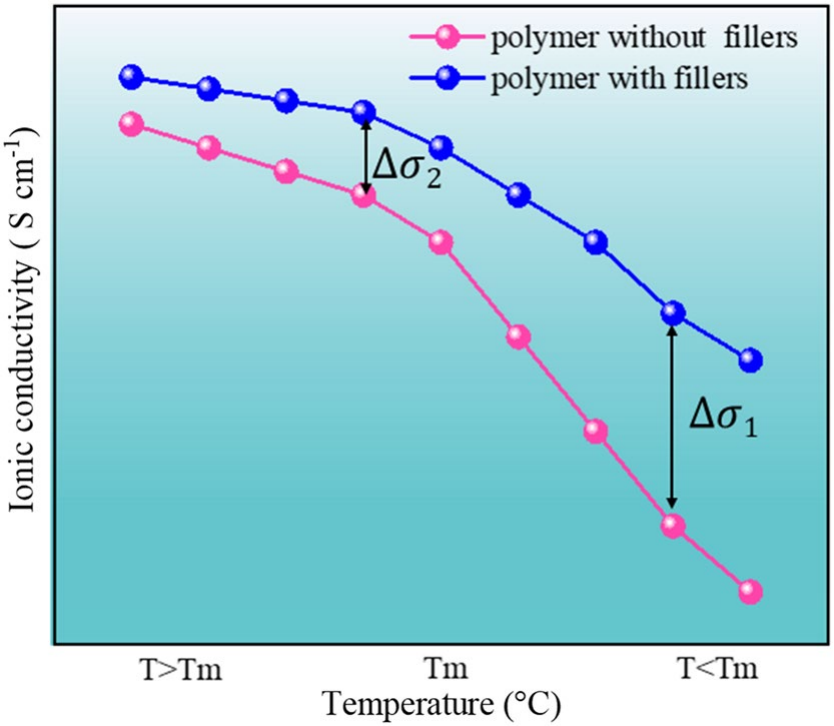
Temperature dependence of ionic conductivity

Therefore, based on the above analysis, we speculate that:

When *T* < Tm, the ionic conductivity of the electrolyte is significantly different for fillers doped or not. As shown in Fig. 18, the ionic conductivity of CPEs is significantly higher than that of SPEs. Even the curve changes relatively slowly. For this, we speculate that the ionic conductivity of CPEs is mainly controlled by the crystallization of the polymer in the low-temperature region. When the filler is doped, the crystalline structure of the polymer is disrupted allowing an additional region for ion conductivity.

Notably, Lewis acid–base interactions at the filler–polymer interface are also present. At this point, the lithium-ion transport behavior is quite complex and governed by several factors. Therefore, ∆σ_1_ is a combination of the inhibition of polymer crystallization by the filler and the Lewis acid–base interactions. When *T* > Tm, we roughly assume that the polymer is completely in the amorphous state. At this moment, the thermal motility of the polymer chains is consistent under the same temperature conditions. When the filler is incorporated, we find that the ionic conductivity of the CPEs is elevated compared to that of the SPEs. It can be approximated that ∆*σ*_2_ is the contribution of Lewis acid–base interactions at the filler–polymer interface.

However, we found that current research regarding Lewis acid–base interactions is generalized. We need to clarify the types of Lewis acid–base interactions, such as electrostatic interactions, van der Waals forces, hydrogen bonds, π-π interactions, etc. Even in CPEs, it should be fully understood which components are the Lewis acids or Lewis bases. In addition, the effects of the same type of inert filler (Al_2_O_3_, SiO_2_, TiO_2_, Ba_2_TiO_3_, etc.) or active filler (LLZTO, LLZO, LATP) on the ionic conductivity, t_Li_^+^, etc., of CPEs still need to be further investigated. This kind of research is important for finding the best-performance inorganic fillers for the application of CPEs.

Furthermore, the impact of the interface compatibility needs to be considered. In recent years, many bulk phase problems, such as the ionic conductivity and t_Li_^+^, have been greatly improved with the increasing research works on CPEs. However, in general, the diffusion of ions at the electrode–electrolyte interface depends on the interfacial contact. Thus, the electrode–electrolyte interface needs to be focused on. On the anode side, the cell is prone to uneven lithium deposition and dendrite growth during lithium embedding and delithiation at high current densities. The ability to resist high voltage on the cathode is the key for the electrolyte to be applied in high energy density batteries. Thus, the chemical, electrochemical, mechanical and thermal stability of the electrode–electrolyte interface becomes another bottleneck in the development of SSLBs. For the electrode–electrolyte interface, CPEs need to possess the following properties:

1. Adhesion. To minimize the interfacial resistance caused by physical contact, the solid-state electrolyte must have good adhesion to the electrode. This may be accomplished by adding some additives, such as plasticizers or liquid electrolytes. However, the amount of plasticizer must be strictly controlled. Otherwise, the mechanical strength of the solid-state electrolyte will be reduced, which can be fatal to the long cycle life of the battery.
2. Efficient and uniform ion transport channels. The uneven deposition of lithium ions on the anode can lead to lithium dendrites, which can threaten the safety of the battery. On the one hand, the space for dendrite growth is reduced by decreasing the physical spaces between the lithium metal and the electrolyte. On the other hand, the uniform deposition of lithium ions is induced by regulating the electrolyte bulk phase. There are two approaches: one is to establish fast and uniform ion transport pathways in the electrolyte to accelerate ion transport and reduce the inhomogeneous charge distribution at the anode. Some ceramic components with different morphologies, such as 3D frameworks, nanowires and nanosheets, can accelerate ions transport. Among them, 3D frameworks and nanowires are of interest because of their long-range continuous ion conduction channels. Second, doping the electrolyte with some low-potential elements is another effective method for inducing uniform lithium deposition.
3. High-pressure compatibility. Most solid-state electrolytes easily decompose when in contact with electrode materials. The interfacial stability of the cathode can be enhanced by changing the HOMO of the polymer through Lewis acid–base interactions. Some inorganic fillers (Al_2_O_3_, etc.) with small molecule plasticizers (SN, etc.) are suitable candidates.
